# Habitat of the endangered salt marsh harvest mouse (*Reithrodontomys raviventris*) in San Francisco Bay

**DOI:** 10.1002/ece3.5860

**Published:** 2020-01-07

**Authors:** Bruce G. Marcot, Isa Woo, Karen M. Thorne, Chase M. Freeman, Glenn R. Guntenspergen

**Affiliations:** ^1^ U.S. Forest Service Pacific Northwest Research Station Portland OR USA; ^2^ U.S. Geological Survey Western Ecological Research Center Moffett Field CA USA; ^3^ U.S. Geological Survey Western Ecological Research Center Davis CA USA; ^4^ U.S. Geological Survey Patuxent Wildlife Research Center Laurel MD USA

**Keywords:** Bayesian network models, California vole, habitat use, *Microtus californicus*, occupancy, *Reithrodontomys raviventris*, salt marsh harvest mouse, San Francisco Bay, San Pablo Bay, small mammal assemblage

## Abstract

Understanding habitat associations is vital for conservation of at‐risk marsh‐endemic wildlife species, particularly those under threat from sea level rise. We modeled environmental and habitat associations of the marsh‐endemic, Federally endangered salt marsh harvest mouse (*Reithrodontomys raviventris*, RERA) and co‐occurrence with eight associated small mammal species from annual trap data, 1998–2014, in six estuarine marshes in North San Francisco Bay, California. Covariates included microhabitat metrics of elevation and vegetation species and cover; and landscape metrics of latitude–longitude, distance to anthropogenic features, and habitat patch size. The dominant cover was pickleweed (*Salicornia pacifica*) with 86% mean cover and 37 cm mean height, and bare ground with about 10% mean cover. We tested 38 variants of Bayesian network (BN) models to determine covariates that best account for presence of RERA and of all nine small mammal species. Best models had lowest complexity and highest classification accuracy. Among RERA presence models, three best BN models used covariates of latitude–longitude, distance to paved roads, and habitat patch size, with 0% error of false presence, 20% error of false nonpresence, and 20% overall error. The all‐species presence models suggested that within the pickleweed marsh environment, RERA are mostly habitat generalists. Accounting for presence of other species did not improve prediction of RERA. Habitat attributes compared between RERA and the next most frequently captured species, California vole (*Microtus californicus*), suggested substantial habitat overlap, with RERA habitat being somewhat higher in marsh elevation, greater in percent cover of the dominant plant species, closer to urban areas, further from agricultural areas, and, perhaps most significant, larger in continuous size of marsh patch. Findings will inform conservation management of the marsh environment for RERA by identifying best microhabitat elements, landscape attributes, and adverse interspecific interactions.

## Graphical abstract

We modeled environmental and habitat associations of federally endangered salt marsh harvest mouse (SMHM) and co‐occurrence with eight associated small mammal species from annual trap data, 1998–2014, in six estuarine marshes in North San Francisco Bay, California. Within the pickleweed marsh environment, SMHM are mostly habitat generalists, with substantial habitat overlap with California voles and other species. SMHM selected for higher marsh elevation, greater plant cover, and large marsh patches.

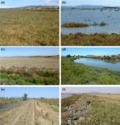

## 1 INTRODUCTION

Tidal marshes constitute a small fraction of land area but typically provide diverse habitat for a unique suite of wetland vertebrate species and contribute disproportionately to regional biodiversity (Greenberg, Maldonado, Droege, & McDonald, 2006; Wiest et al., 2016; Zedler, Callaway, & Sullivan, 2001). Tidal marshes are becoming increasingly vulnerable to a variety of threats and stressors (Kintisch, 2013), including inundation from high tides caused by local sea level rise and storms (Craft et al., 2009; Field, Gjerdrum, & Elphick, 2016; Kirwan & Temmerman, 2009; Thorne et al., 2018), differential response of wetland plants to flooding (Janousek et al., 2016; Kirwan & Guntenspergen, 2015), changes in vegetation productivity from local warming (Maegonigal et al., 2016), incursion by invasive species (Winder, Jassby, & MacNally, 2011), coastal erosion (Kane, Fletcher, Frazer, Anderson, & Barbee, 2015), urban development (Enwright, Griffith, & Osland, 2016; Shellhammer, 1989), pollution (McCann et al., 2017), and other factors. All such changes can impact suitability of tidal marsh habitat for the variety of associated wildlife species, especially habitat specialist and endemic species (Takekawa et al., 2006; Thorne, Takekawa, & Elliott‐Fisk, 2012; Torio & Chmura, 2015). Due to these direct and indirect human impacts, many marsh wildlife species are listed as endangered or threatened pursuant to the U.S. Endangered Species Act. It is likely that many changes to ecosystems caused by shifting climate patterns have not been recognized, but habitats can respond quickly and drastically in unpredictable ways, leading to substantial uncertainty about impacts to endemic wildlife. Given this, uncertainty defining habitat characteristics is growing increasingly important for the conservation of biodiversity, especially for at‐risk species.

**Figure 1:**
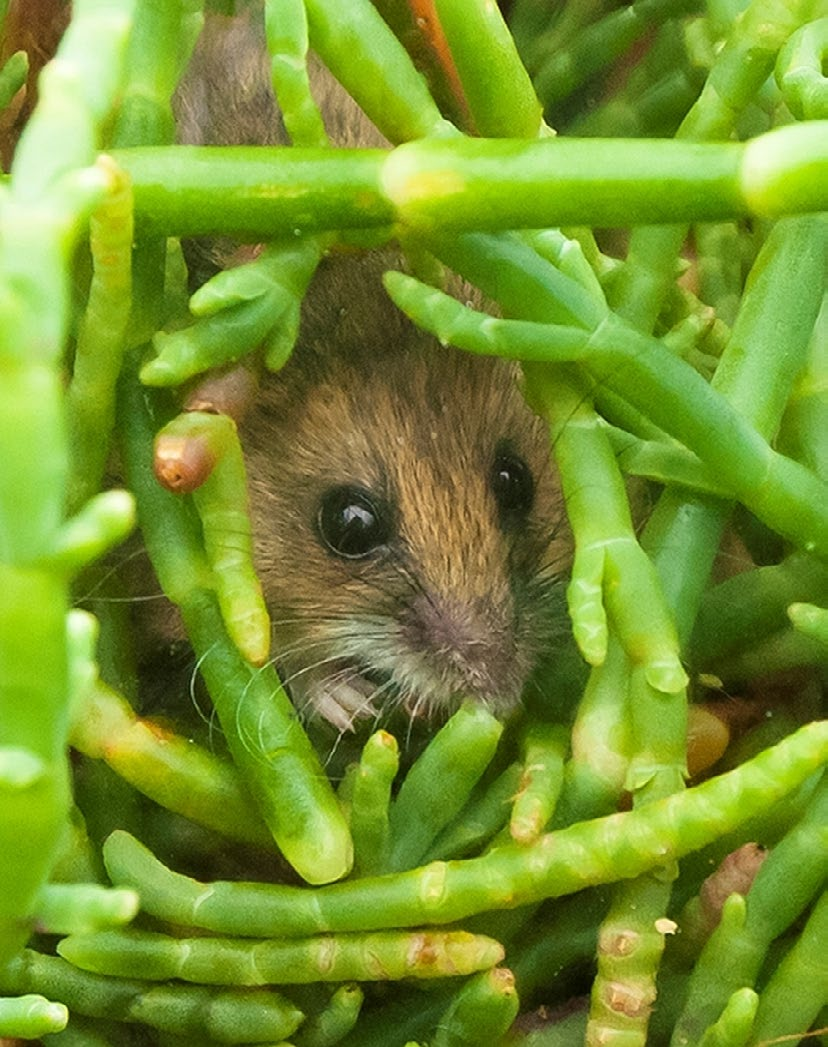
Salt marsh harvest mouse (*Reithrodontomys raviventris*) in pickleweed (*Salicornia pacifica*) habitat. (Photo used by permission, Judy Irving © Pelican Media)

Many resource management agencies have established a habitat‐based approach, rather than an individual species approach, for recovery of marsh endemic species (DOI, 2013; Goals Project, 2015; USFWS, 2013), making it all the more important to understand habitat relationships of at‐risk species presumed to be provided by a habitat‐based approach. Our study focused on the salt marsh harvest mouse (*Reithrodontomys raviventris*, RERA, Figure 1), federally‐, state‐, and IUCN‐listed as endangered (CDFG, 2015; USFWS, 2013; http://www.iucnredlist.org/species/19401/22385344). RERA is endemic to the wetlands of the San Francisco Bay (SFB), California, where marsh plant communities and obligate wildlife are at risk of habitat loss from local sea level rise over the coming decades (Thorne, Buffington, Elliott‐Fisk, & Takekawa, 2015; Veloz et al., 2013).

RERA currently occupies <25% of its historic range because of habitat fragmentation and loss, with >80% loss of historic wetland habitat in SFB (Statham et al., 2016). The northern subspecies (*Reithrodontomys raviventris halicoetes*) is found along San Pablo and Suisun Bays, and the southern subspecies (*R. r. raviventris*) is found in South SFB with the subspecies divide somewhere in Central Bay (Shellhammer, 1989; Statham et al., 2016; USFWS, 2013). However, detailed habitat associations of both subspecies are not well understood. Smith, Riley, Barthman‐Thompson, Woo, et al. (2018) reviewed the RERA literature to date. Smith, Riley, Barthman‐Thompson, Statham, et al. (2018) recommended research priorities to further RERA recovery, including the need for data synthesis to assess regional habitat associations and species interactions. On this basis, we collated several monitoring datasets to address this priority research need.

Several independent monitoring efforts have been conducted to assess SMHM abundance with varying sampling design and effort, very low SMHM captures, and lack of marking individual captures, such that traditional occupancy models would not be appropriate. Therefore, we used a probabilistic analysis approach with Bayesian network (BN) modeling to determine RERA correlates with environmental and habitat parameters, and to evaluate relationships with other sympatric small mammal species.

Bayesian network models link variables with probabilities calculated using Bayes' Theorem (Korb & Nicholson, 2010; Koski & Noble, 2011) and are relatively robust to zero inflated data and collinearity. We analyzed trap data and associated vegetation, patch (elevation and inundation), and landscape covariates, including presence of other small mammal species, to determine habitat associations of RERA. Explicitly, we aimed to assess (a) microhabitat and landscape attributes determining RERA presence, and (b) if RERA presence was associated with other small mammal species. We developed all BN models from empirical field and spatial data. This modeling approach can be used to project impacts on RERA from future changes in habitat conditions, disturbances, and climate change, and to inform management of these tidal marsh environments to facilitate species recovery and conservation.

## 2 METHODS

### 2.1 Study area

The SFB ecosystem in the central coast of California has a Mediterranean climate with warm dry summers and cool rainy winters. North SFB (38°08′N, 122°24′W), comprised of San Pablo and Suisun Bays, receives most of its freshwater from the Sacramento and San Joaquin Rivers in the form of summer snowmelt from the Sierra Nevada mountains. This region has a mixed semidiurnal tide with a mean tide range of 1.17–1.63 m and a mean spring tide range of 1.57–2.09 m (Parker, Callaway, Schile, Vasey, & Herbert, 2012). Most tidal marshes are found above mean tide level (MTL, defined as the arithmetic mean of mean high water and mean low water; Parker et al., 2012) whereas marsh plain elevations are closer to or above mean high water (MHW, defined as the average of all the high water heights observed over 19 years; Takekawa et al., 2013) (https://shoreline.noaa.gov/glossary.html). The marsh plain is dominated by pickleweed (*Salicornia pacifica*, syn. *Sacrocornia pacifica*, formerly *Salicornia virginica*; Calflora, 2018), and the lower marsh area is dominated by *Spartina foliosa*, a native of the region.

Examples of local marsh inhabitants are salt marsh common yellowthroat (*Geothlypis trichas sinuosa*), San Pablo song sparrow (*Melospiza melodia samuelis*), and San Pablo vole (*Microtus californicus sanpabloensis*), state‐listed species of special concern; California black rail (*Laterallus jamaicensis coturniculus*), a state threatened species; and California Ridgway's rail (*Rallus obsoletus*, formerly clapper rail, *Rallus longirostris obsoletus*). However, RERA is the only mammal species in the world that is entirely endemic to coastal marshes (Greenberg & Maldonado, 2006) and is found only in tidal marshes in SFB (Figure 1). It currently occupies <25% of its historic range because of habitat fragmentation and loss, with >80% loss of historic wetland habitat in SFB (Statham et al., 2016). The northern subspecies (*R. r. halicoetes*) is found along San Pablo Bay, and the southern subspecies (*R. r. raviventris*) is found in South SFB with the subspecies divide somewhere in Central Bay (Shellhammer, 1989; Statham et al., 2016; USFWS, 2013).

### 2.2 Field trapping

**Figure 2:**
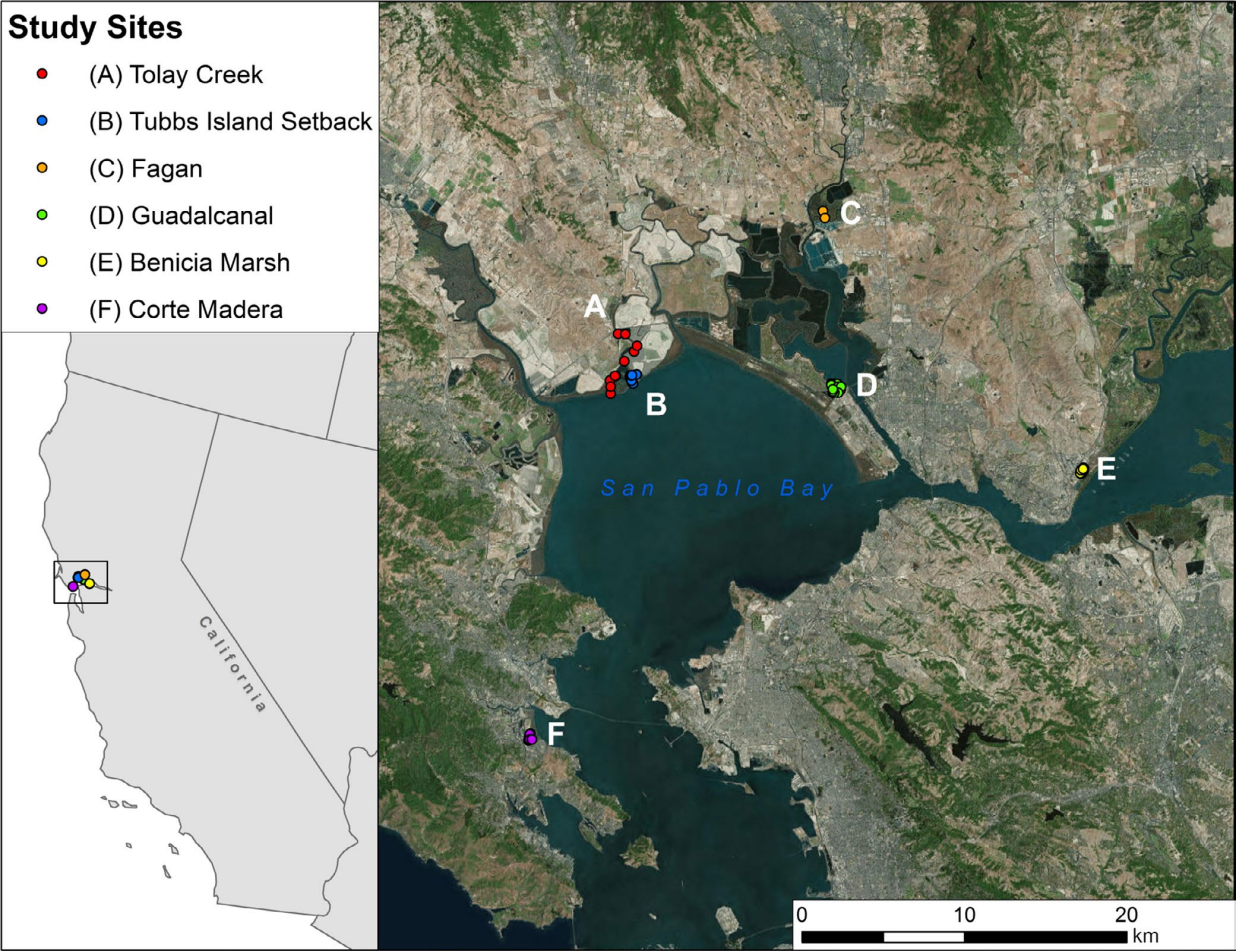
Locations of small mammal trap sites around North San Francisco Bay, California

Small mammal surveys were conducted as separate monitoring efforts, and for these analyses results were collated from estuarine marsh study sites in San Pablo Bay (Fagan, Guadalcanal, Tolay Creek, and Tubbs Island Setback), Grizzly Bay (Benicia‐Martinez Marsh), and Central Bay (Corte Madera) (Figure 2). Over the course of the study (1998–2014), a total of 12,405 trap nights were conducted (Table 1). Sherman live traps (H.B. Sherman Traps, Inc.) were placed in three trap layout patterns (Table 2): grid, transect, and random placement. Grids varied in size, covering arrays of 5 × 5, 5 × 10, 7 × 7, and 2 × 25 traps, depending on local conditions and marsh area. Where local marsh conditions narrowed, not providing adequate area for a grid, one to three transects were used, each consisting of ten traps per transect. Random placements of traps were used only in the Tubbs Island Setback site because tidal marsh habitat and the upland transition zone were narrow with variable width such that the random placement allowed better coverage by which to associate RERA captures to habitat. No one site used all three trap layout patterns, and all sites used the grid or transect patterns except for Tubbs Setback (Table 2).

**Table 1:** Number of trap nights by year and location (see Figure 2)

Year	Benicia‐Martinez Marsh	Corte Madera	Fagan	Guadalcanal	Tolay Creek	Tubbs Island Setback	Total
1998	0	0	0	0	600	0	600
1999	0	0	0	0	2,270	0	2,270
2000	0	0	0	0	634	0	634
2001	0	0	0	0	330	0	330
2002	0	0	0	0	660	0	660
2003	0	0	0	225	660	360	1,245
2004	0	0	0	0	618	360	978
2005	0	0	0	195	588	360	1,143
2006	225	0	0	225	225	360	1,035
2007	225	0	0	225	300	360	1,110
2008	195	0	0	0	225	360	780
2009	0	0	0	0	225	0	225
2010	0	0	0	0	225	360	585
2011	0	0	300	0	0	360	660
2014	0	150	0	0	0	0	150
Total	645	150	300	870	7,560	2,880	12,405

**Table 2:** Number of trap nights by site and trap layout pattern

Site	Grid	Random	Transect	Total
Benicia‐Martinez Marsh	225	0	420	645
Corte Madera	0	0	150	150
Fagan	300	0	0	300
Guadalcanal	300	0	570	870
Tolay Creek	6,840	0	720	7,560
Tubbs Island Setback	0	2,520	360	2,880
Total	7,665	2,520	2,220	12,405

Traps were spaced at 10‐m intervals following Jones, McShea, Conroy and Kunz (1996). This spacing was also consistent with findings from Bias and Morrison (1999) who reported that RERA moved short distances (mean 11.9 m) between consecutive 2‐hr telemetry observations, and that RERA home ranges averaged just 2,133 m^2^. Traps were initially deployed twice a year in spring and summer at Tolay Creek to capture a range of reproductive conditions. At other sites, traps were deployed once a year in late summer or early fall to maximize the capture of juveniles who would have been born in the spring of the same year. Although individual RERA movements patterns were generally highest in summer (June) and lowest in early winter (November), movements of pregnant females were low more consistently throughout the seasons (Bias & Morrison, 1999). Therefore trap distance was kept at a 10‐m interval regardless of season.

Each trapping session consisted of three consecutive nights (with limited exploratory sampling conducted for four nights at Tolay Creek to increase detection of RERA). Traps were baited with a mix of crushed walnuts, birdseed, and mealworm (for insectivorous shrews) and opened in the evenings, checked each morning at sunrise, and closed during the day.

Polyester batting was placed within each trap to keep small mammals warm. Wooden shingles were placed on top of each trap to protect captured animals from exposure. Species identification, sex, age, mass (mg), reproductive condition, body length, tail length, and presence of wounds or parasites were recorded for all individuals. Reproductive condition in males was characterized by presence and development of the testes. Reproductive condition in females was characterized by the presence and development of mammaries and whether the animal was pregnant. Animals captured and identified to the genus *Reithrodontomys* also included records of tail width 20 mm from the base of the tail, hind foot length, ear length, venter coloration of tail and belly, bicoloration of tail, and behavior (e.g., aggressiveness). Individuals were marked by clipping fur with small scissors to identify recaptures.

### 2.3 Microsite and patch covariates

We recorded microsite and patch conditions in terms of vegetation, topography, and inundation patterns at trap locations. Each trap location was defined by a set of covariates including trap data, location, plant species, elevation, distance to natural or anthropogenic features, and marsh patch size (Appendix S1: Table S1; see Metadata S1 for the list and definitions of covariates), as follows.

#### 2.3.1 Vegetation

**Figure 3:**
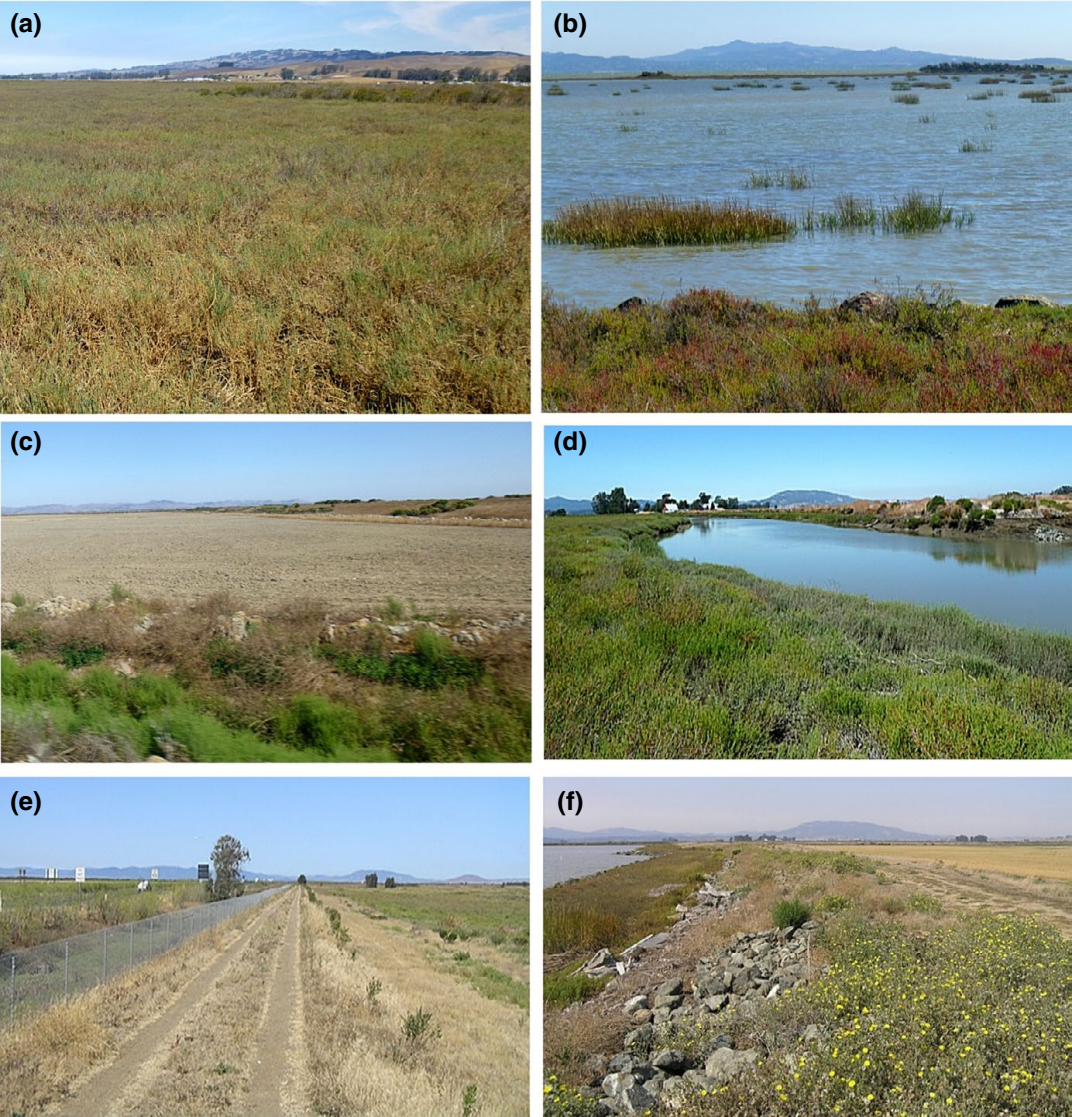
Examples of North San Francisco Bay salt marsh environments (see Figure 1 for place name locations). (a) Pickleweed salt marsh, Tolay Creek. (b) San Pablo Bay, Tubbs Setback. (c) Fallow agricultural hay field, from levee, Tubbs Setback. (d) Channel > 3m wide. (e) Road on levee. (f) Wetland, levee, and agricultural field. (Photos a–c by Bruce G. Marcot, d–f by Isa Woo)

We visually assessed percent cover of each vascular plant species within a 0.25 m^2^ vegetation quadrat centered on each trap and measured the mean and maximum height (to nearest cm) of each species. In wetland restoration sites, habitat at each trap was described using the closest and the second‐closest vegetation quadrats for Tolay Creek (Bias et al., 2006), Tubbs Setback (Woo, Takekawa, Rowan, Gardiner, & Block, 2007), Guadalcanal (Woo, Takekawa, Gardiner, Dembosz, & Bishop, 2009), and Benicia‐Martinez (Woo, Bishop, & Takekawa, 2010). For our covariate data set, we identified from the vegetation plots the plant species or other cover categories having the three highest individual cover percentages, maximum heights, and average heights; we used all these covariates in the initial modeling, discussed below. Other cover categories recorded and use in initial modeling included presence and percent cover of green algae, brown algae, bare ground, litter, dead plants standing, and dead plants not standing. Total plant cover in a quadrat could exceed 100% due to vegetation layering. We followed Jepson Flora Project (2018) for vascular plant nomenclature. See Figure 3 for examples of vegetation and environmental conditions.

#### 2.3.2 Topography

We measured topographic variation at each site by surveying marsh surface elevation along transects perpendicular to the major tidal sediment source, with survey points taken every 12.5 m, and transects separated by 50 m using a Real Time Kinematics (RTK) GPS (Leica Geosystems Inc.). We used the Geoid09 model to calculate orthometric heights from ellipsoid measurements (m, NAVD88; North American Vertical Datum of 1988) and projected all points to NAD83 UTM Zone 10 using Leica GeoOffice v7.0.1 (Leica Geosystems Inc.). We then combined the elevation survey data to create a digital elevation model (DEM) at each site in ArcGIS 10.2.1 Spatial Analyst (ESRI 2011) with exponential ordinary kriging methods (5 × 5 m cell size) after adjusting model parameters to minimize the root‐mean‐square error (RMS). Using the DEM data, we were then able to assign a specific elevation to each trap location.

#### 2.3.3 Inundation

To determine inundation patterns and calculate site‐specific tidal datums, we used a mix of available local data sources. We deployed water level data loggers (Model 3001; Solinst Canada Ltd.) at Corte Madera, Tolay Creek and Tubbs Island Setback Marshes (Takekawa, Thorne, Buffington, & Freeman, 2014; Takekawa et al., 2013). For other sites (Benicia‐Martinez, Fagan, Guadalcanal), we used data from the nearest NOAA tidal station. Sites with locally collected data had one or two loggers that were placed at the mouth and upper reaches of second‐order tidal channels to capture high tides and determine seasonal inundation patterns. Water level readings were collected every 6 min starting on the date of deployment and continued for at least one year to calculate tidal datums for each trapping location.

We used data from the lowest elevation logger at each site to develop local hydrographs and inundation rates, and we surveyed loggers with RTK GPS at the time of deployment and at each data download to correct for any vertical movement and to reference water levels to NAVD88 elevations. All raw water level data were corrected with a local time series of barometric pressure. For Solinst loggers, we deployed independent barometric loggers (Model 3001; Solinst Canada Ltd.). We used water level data to estimate local tidal datums for all sites using procedures outlined in the NOAA Tidal Datums Handbook (NOAA 2003), and calculated only local mean high water (MHW) and mean higher high water (MHHW) because the loggers were positioned in the intertidal and therefore could not be used to compute lower datums. We then assigned MHW and MHHW values to each trap location based on the nearest local water monitoring location and its associated local tidal datums (Figure 2).

### 2.4 Statistical modeling

#### 2.4.1 Correlation among variables

We first conducted bivariate Pearson correlations among the covariates to determine which variables may be highly correlated so as to exclude them from further modeling. Although the BN models we used are more tolerant of multicollinearity among covariates than are frequentist multivariate models (Pawson, Marcot, & Woodberry, 2017), it is still useful to narrow the set of covariates to reduce unnecessary model complexity.

#### 2.4.2 Bayesian network structure

We chose BNs as the modeling construct because of several key advantages of this approach over frequentist approaches: BN models can well handle missing data, can incorporate and display uncertainties in frequency distributions of values of variables, can account for correlations among covariates, can produce usable results with uneven sample sizes of covariates, and other advantages (Oniśko, 2008; Pawson et al., 2017). BN models can also be used to incorporate data from different sources, such as the variety of RERA trap layout patterns and trapping intensities in the current study.

Each BN model was developed in Netica^®^ (vers. 6.06; Norsys, Inc.) using the per‐trap results as a case file database (available as Metadata S1). Covariates were incorporated by use of the "add case file nodes" in Netica, which converts the covariates into BN variables (network nodes). Continuous variables were discretized (cutoff values of "bin" states were identified) into exclusive states using Netica's "modify/auto discretize" function, specifying no more than five states per variable so as to make the sizes of the conditional probability tables in the final models tractable and allowing sufficient sample sizes (approximately 20 or greater) in each state.

Next, we induced BN model structures from the case file by using the tree‐augmented network (TAN) algorithm (Friedman, Geiger, & Goldszmidt, 1997; Jiang, Cai, Wang, & Zhang, 2012). This identifies key linkages among the BN variable nodes by using a modified naïve Bayes learning rule. That rule does not presuppose any specific dependencies among the variables in the model, and it connects variables according to their correlations once the dependent variable is initially identified by the user. In general, a naïve BN is structured with links extending from the response variable to covariates, representing the degree to which variation in the response variable is explained by the covariates. The linkages also connect any covariates to each other if they are still correlated in the network structure even after some variables were initially excluded for highest correlation. Values of all unconditional and conditional probability tables (CPTs) in the model then were specified from the case file by using Netica's "incorporate case file" learning algorithm, that calculates the probabilities from frequency occurrences in the case file.

We created a series of BN models based on various subsets of the selected covariates, using the two response variables of (a) presence of RERA in trap results, and (b) capture results of all small mammal species including RERA. We used the second response variable of all small mammal species to determine if the presence of other small mammal species could help account for presence or nonpresence of RERA, thus potentially improving model performance. The model‐construction procedure—developing BN models entirely induced from the field data case file—was repeated for each response variable of RERA presence and all‐species trap outcomes. For each of these two response variable outcomes, we developed 19 variants of BN models, thus 38 total models, to represent different combinations of covariates. We grouped covariates into logical sets representing trap sampling design, location (latitude–longitude), vegetation and cover, elevation, distance to natural or anthropogenic features, and marsh patch size, and then developed BN models using variables drawn from one or more of those sets (Table 3).

**Table 3:** Variants of Bayesian network (BN) models using combinations of covariates

Model no.	Covariate (predictor variable) sets used						
	C1. Trap data	C2. Latitude–longitude	C3. Vegetation & cover^a^	C4. Vegetation & cover^b^	C5. Elevation	C6. Distance	C7. Patch size
1, 20	X						
2, 21		X					
3, 22			X				
4, 23				X			
5, 24					X		
6, 25						X	
7, 26							X
8, 27					X	X	
9, 28						X	X
10, 29					X		X
11, 30			X		X	X	
12, 31			X			X	X
13, 32			X		X		X
14, 33			X		X	X	X
15, 34	X		X		X	X	
16, 35	X		X			X	X
17, 36	X		X		X		X
18, 37	X		X		X	X	X
19, 38				X	X	X	X

Note

These variants were applied to BN models with salt marsh harvest mouse presence response (model numbers 1–19) and BN models with all small mammal species response (model numbers 20–38). Covariates are described in Table S1.1.

a C3 Vegetation and Cover covariates pertain to the presence, percent cover, maximum height, and average height of the most dominant plant species within the closest vegetation plot to the trap.

b C4 Vegetation and Cover covariates include the C3 Vegetation and Cover covariates and also the same for the second and third most dominant plant species within the closest vegetation plot to the trap.

#### 2.4.3 BN model accuracy, complexity, selection, and sensitivity

We identified the best‐performing models as those with low complexity and high accuracy (low overall prediction error. and low Type I error of false positive). The overall aim of denoting model calibration accuracy and complexity was to provide information by which to choose best models, which would be those with highest classification accuracy (lowest error) and lowest model complexity, akin to use of Akaike information criterion (AIC) metrics in frequentist statistical modeling (Akaike, 1973) that is strictly not applicable in BN modeling. Selection of best Bayesian models can be a somewhat subjective process (Hooten & Hobbs, 2015). Despite the diversity of model performance metrics, there are no agreed‐upon methods for Bayesian model selection. Thus, we present here accuracy and complexity outcomes of all models as an objective procedure.

We then tested each BN model variant for calibration accuracy by calculating classification error rates ("confusion tables" sensu Kohavi & Provost, 1998) using the trap data case file. This entailed running each BN model for each case in the case file and determining if the model would have resulted in a dominant probability outcome that is the same as the known case outcome (presence of RERA, or presence of a specific mammal species).

We partitioned calibration classification error rates into Type I (false positive) and Type II (false negative) errors. We calculated Type I errors as the number of cases when the model predicted presence of RERA (or some other species) when the actual trapping result was RERA nonpresence (or another species outcome), divided by the total number of cases when the model predicted presence (thus including when the actual outcome was presence). We calculated Type II errors as the number of cases when the model predicted nonpresence of RERA (or some other species) when the actual trapping result was presence (or another species outcome), divided by the total number of cases when the model predicted nonpresence (thus including when the actual outcome was nonpresence). We used these calculations and separated the error types because they carry very different management implications depending on trap results, as we discuss below.

We also calculated values of spherical payoff for each model variant. Spherical payoff is an index of model calibration performance that compares model outcomes to known outcomes across a case file, averaging the probability of each predicted state and each known state (for calculations, see Marcot, 2012; Morgan & Henrion, 1990). Values of spherical payoff range [0,1] where 1 denotes best performance (lowest classification error) and 0 denotes the worst. We include spherical payoff because it provides complementary information to, and can be poorly correlated with, overall confusion error rate (Marcot, 2012).

For each BN model variant, we also denoted three measures of model complexity: number of nodes (variables), number of links, and number of probability values. We used these three measures of complexity because, depending on model structures resulting from the model‐building algorithms, they are not necessarily significantly correlated (Marcot, 2012).

We then conducted 4‐fold cross‐validation tests of model validity, on the selected best models, following k‐fold procedures described in Marcot (2012) and as used in Bayesian modeling by Broms, Hooten, and Fitzpatrick (2016), Pawson et al. (2017), and others. The purpose of conducting cross‐validation was to determine the degree of fit or overfit of each model; a BN model is likely overfit if it has high calibration accuracy (low classification confusion error) but low cross‐validation prediction accuracy. The final selection(s) of best model(s) should have high calibration accuracy, low complexity, and high cross‐validation prediction accuracy.

Lastly, we conducted sensitivity tests of the best models to determine the degree to which the predicted occupancy probabilities were sensitive to uncertainty about each covariate. We used sensitivity analysis procedures and metrics detailed in Marcot (2012) entailing calculations of entropy reduction which depict incremental responses of an outcome variable given incremental changes in each covariate. Results are useful for comparing relative sensitivity among covariates within a model, and relative sensitivity of a given covariate among models.

#### 2.4.4 Habitat relationships

We used the trap results, data on environmental conditions at each trap sites, and the BN models, to evaluate habitat conditions of RERA. We also compared habitat conditions of RERA to those of other small mammals, based on BN model comparisons. We used results of unpaired *t* tests and *F* tests to compare covariate associations between RERA the other small mammal species captured during this study.

## 3 RESULTS

### 3.1 Site attributes

Among all sites, trap locations in Tubbs Island Setback had the highest average elevation above MTL and the highest average marsh plain elevation above MHW and MHHW, and Tolay Creek had the lowest. Benicia‐Martinez Marsh and Corte Madera were furthest from agricultural lands but among the closest to other anthropogenic features; Tubbs Island Setback was furthest from roads and, with Tolay Creek, also furthest from urban areas; and Fagan was furthest from the bay and from levees (Figure S1.2). Tolay Creek had the largest average habitat patch size, and Guadalcanal had the largest extended habitat patch size not impeded by barriers or channels >3 m wide (Figure S1.3).

### 3.2 Plant species

Among all trap locations, at least 20 vascular plant species were recorded (Table S1.2). Across all trap sites, pickleweed was the most dominant cover category (54% of trap sites), followed by bare ground (24%), salt grass (9%), alkalai heath (5%), and other species and categories (<3% each).

### 3.3 Environmental and vegetation covariates

Of the 78 Pearson correlations *r* among 13 continuous environmental covariates (elevation, distance, and patch size variables), nine were statistically significant (*p* < .05) with |*r*| > 0.75 (Table S1.3). From these results, we eliminated three covariates (MHHW, distance to closest urban area and distance to closest agriculture), leaving the remaining 10 to include in the BN models in various combinations with low correlation. The 12 vegetation covariates were not part of the correlation analysis due to sparsity of these data (low numbers of plots in which all variables were present), but we included all of them in the BN models.

### 3.4 Small mammals

Field trapping resulted in capture of nine species of small mammals (Table 4) over 12,405 trap nights, with 73% of trap nights resulting in no captures. The most frequently captured species were California vole (*M. californicus*, MICA), RERA, house mouse (*Mus musculus*), and deer mouse (*Peromyscus maniculatus*). MICA captures in this study overlap the geographic region and habitat of the San Pablo subspecies (*M. californicus sanpabloensis*, a California State Mammal of Concern); however, subspecies identification was not possible for our live captures. RERA constituted 20% of all captures and 5% of all trap nights. All RERA captures were of the northern subspecies, *R. r. halicoetes*. There were no RERA captures at Corte Madera and a single RERA capture at Benicia‐Martinez Marsh. RERA capture rates (n/trap night) were highest at Fagan Marsh and Tolay Creek study sites (Table 5, Figure 2).

**Table 4:** Results of species trap captures over a total of 3,339 trap nights

Response variable		No. trap nights^a^
Presence results		
RERA	Presence of salt marsh harvest mouse, *Reithrodontomys raviventris*	669 (20%)
NOTRERA	Salt marsh harvest mouse not present (treated as absence of capture)	2,670 (80%)
Species‐specific results		
MICA	California vole, *Microtus californicus*	1,565 (47%)
MUMU	House mouse, *Mus musculus*	555 (17%)
PEMA	Deer mouse, *Peromyscus maniculatus*	433 (13%)
RANO	Norway rat, *Rattus norvegicus*	14 (<1%)
RARA	Black rat, *Rattus rattus*	1 (<1%)
REME	Western harvest mouse, *Reithrodontomys megalotis*	37 (1%)
RERA	Salt marsh harvest mouse, *Reithrodontomys raviventris*	669 (20%)
SOOR	Ornate shrew, *Sorex ornatus*	65 (2%)

a Additionally were 9,066 trap nights with no captures (73% of all trap nights including the 3,339 with captures).

**Table 5:** Capture results of small mammals by study site: number of trap nights with individual captures and trap outcomes (outcomes per 100 trap nights in parentheses)

Species	Benicia‐Martinez Marsh	Corte Madera	Fagan	Guadalcanal	Tolay Creek	Tubbs Island Setback	Total
MICA	0 (0.0)	0 (0.0)	16 (5.3)	2 (0.2)	1,437 (19.0)	110 (3.8)	1565 (12.6)
MUMU	22 (3.4)	1 (0.7)	10 (3.3)	156 (17.9)	211 (2.8)	155 (5.4)	555 (4.5)
PEMA	0 (0.0)	0 (0.0)	0 (0.0)	0 (0.0)	181 (2.4)	252 (8.8)	433 (3.5)
RANO	0 (0.0)	0 (0.0)	0 (0.0)	0 (0.0)	4 (0.1)	10 (0.4)	14 (0.1)
RARA	0 (0.0)	0 (0.0)	0 (0.0)	0 (0.0)	0 (0.0)	1 (0.03)	1 (0.01)
RE	0 (0.0)	3 (2.0)	0 (0.0)	0 (0.0)	0 (0.0)	0 (0.0)	3 (0.02)
REME	0 (0.0)	1 (0.7)	0 (0.0)	0 (0.0)	33 (0.4)	0 (0.0)	34 (0.3)
RERA	0 (0.0)	0 (0.0)	22 (7.3)	19 (2.2)	546 (7.2)	82 (2.9)	669 (5.4)
SOOR	0 (0.0)	0 (0.0)	1 (0.3)	0 (0.0)	63 (0.8)	1 (0.03)	65 (0.5)
All Spp.	22 (3.4)	5 (3.3)	49 (16.3)	177 (20.3)	2,475 (32.7)	611 (21.2)	3,339 (26.9)
TRAP	623 (96.6)	145 (96.7)	251 (83.7)	693 (79.7)	5,085 (67.3)	2,269 (78.8)	9,066 (73.1)
Total^a^	645 (100)	150 (100)	300 (100)	870 (100)	7,560 (100)	2,880 (100)	12,405 (100)

Note

See Table 4 for species codes; TRAP = traps set but no captures resulted.

a Total = sum of All Spp. and TRAP results.

### 3.5 Bayesian network models

We developed 38 variants of BN models consisting of 19 different combinations of covariates, each with two variations of trap‐result response variables: RERA presence, models 1–19; and presence of each small mammal species, models 20–38 (Table 3; Figure S1.4).

Among the RERA presence models (Table 6, Table S1.4), model complexity varied widely among the model variants. Complexity varied from 2 to 23 variables (model nodes), 1–43 links among model nodes, and 12–5,140 probability values. Overall calibration classification error rates varied 19%–47%, with Type I errors varying 0%–100% and Type II errors varying 15%–20%, and spherical payoff ranged from 0.634 to 0.849.

**Table 6:** Results of evaluation of Bayesian network (BN) model complexity and accuracy (calibration performance), using salt marsh harvest mouse presence as the response variable

Model no.	Model complexity			Model accuracy (calibration performance)			
	No. nodes	No. links	No. probs.	Overall confusion error (%)	Type I error (false presence, %)	Type II error (false nonpresence, %)	Spherical payoff
1	6	9	1,400	19%	22%	19%	0.849
2^a^	3	3	62	20%	0%	20%	0.833
3	5	7	272	20%	100%	20%	0.828
4	13	22	902	44%	79%	20%	0.688
5	5	7	170	20%	0%	20%	0.832
6^a^	2	1	12	20%	0%	20%	0.826
7^a^	3	3	62	20%	0%	20%	0.832
8	9	15	370	21%	56%	17%	0.829
9	7	11	262	21%	56%	17%	0.827
10	7	11	280	21%	56%	18%	0.827
11	13	23	682	22%	57%	17%	0.825
12	11	19	572	21%	56%	17%	0.819
13	11	19	612	21%	56%	18%	0.819
14	15	27	792	22%	57%	17%	0.813
15	18	33	4,620	19%	34%	19%	0.840
16	16	29	4,260	19%	33%	19%	0.840
17	16	29	4,100	19%	34%	19%	0.841
18	20	37	5,140	19%	35%	19%	0.839
19	23	43	1,642	47%	76%	15%	0.634

a Selected models that best balance low model complexity with high model accuracy (low error rates).

Among the all‐species presence models (Table 7, Table S1.5), model complexity also varied widely among the model variants. The number of nodes and number of links were the same as with the RERA presence models, but the number of probability values ranged 54–23,130. Overall calibration classification error rates varied 37%–59%, with Type I errors varying 0%–100% and Type II errors varying 15%–20%, and spherical payoff ranged from 0.518 to 0.700.

**Table 7:** Results of evaluation of Bayesian network (BN) model complexity and calibration performance for presence of salt marsh harvest mouse (RERA), using all 9 small mammal species captured as the response variable

Model no.	Model complexity			Model accuracy (calibration performance for RERA)			
	No. nodes	No. links	No. probs.	Overall confusion error (%)	Type I error (false presence, %)	Type II error (false nonpresence, %)	Spherical payoff
20	6	9	6,300	37%	39%	18%	0.700
21	3	3	279	46%	55%	20%	0.624
22	5	7	1,224	46%	100%	20%	0.621
23	13	23	4,959	52%	80%	20%	0.566
24	5	7	765	43%	50%	20%	0.639
25^a^	2	1	54	49%	0%	20%	0.600
26^a^	3	3	279	48%	0%	20%	0.617
27	9	15	1,665	44%	56%	18%	0.628
28	7	11	1,179	43%	55%	20%	0.632
29	7	11	1,260	43%	0%	20%	0.643
30	13	23	3,069	44%	57%	18%	0.618
31	11	19	2,799	43%	56%	17%	0.627
32	11	19	2,754	44%	58%	18%	0.631
33	15	27	3,564	43%	55%	18%	0.612
34	18	33	20,790	37%	35%	19%	0.689
35	16	29	19,170	37%	36%	19%	0.691
36	16	29	18,450	37%	34%	19%	0.691
37	20	37	23,130	37%	34%	19%	0.687
38	23	43	7,704	59%	74%	15%	0.518

a Selected models that best balance low model complexity with high model accuracy (low error rates).

For RERA presence, the best‐performing models were models 2, 6, and 7 (Table 6), and for all‐species presence, these were models 25 and 26 (Table 7). The three best RERA presence models consisted of ≤3 nodes, ≤3 links, and ≤62 probability values, and had 20% overall calibration classification error, 0% Type I error, 20% Type II error, and spherical payoff values >0.82. The two best all‐species presence models consisted of ≤3 nodes, ≤3 links, and ≤279 probability values, and had 48% and 49% overall calibration classification error, 0% Type I error, 20% Type II error, and spherical payoff values ≥0.60.

All best‐performing models were extremely simple with few covariates that adequately predicted RERA outcomes. Model 2 used latitude and longitude (among all study sites, greater RERA presence in sites further east and in middle latitudes); models 6 and 25 used distance to roads (greater RERA presence > 1,000 m from roads); and models 7 and 26 used salt marsh patch size and expanded patch size (greater RERA presence in continuous marsh ≥20 ha that are not impeded by barriers of channels >3 m wide or levees) (see Table S1.1 for covariate definitions).

Models 1 and 20, based on just study site location, sampling time, and sampling method, performed fairly well. However, they were more complex and incurred greater Type I error rates than the best‐performing models. Models 2 and 21, based on just latitude and longitude, fared well. This indicted that, for the general study region, site location is a fair predictor of RERA presence, although these models provided no information on habitat or environmental conditions. However, models 3, 4, 22, and 23, based solely on vegetation, fared very poorly with high Type I errors. Clearly, something other than, or in addition to, vegetation—viz., elevation, distance to roads, and marsh patch size—influenced RERA presence. The remaining models were generally increasingly complex with various mixes of covariate sets, but improvements to calibration error rates were minor at best and not offset by the greatly increased levels of model complexity.

Cross‐validation results (Table S1.6) were essentially the same as the calibration error analyses (Tables 6 and 7), conveying that the best‐performing BN models were not overfit. Sensitivity analyses of best‐performing models (Table S1.7) suggested the following regarding presence of RERA. RERA presence was more sensitive to longitude (easting) than to latitude (northing). Also, presence of all‐species, compared with presence of RERA, was slightly more sensitive to distance to roads and much more sensitive to patch size.

None of the 19 BN models of RERA presence provided a classification confusion error rate of <19%. Four models resulted in no Type I errors of false presence predictions, but those models each incurred a 20% Type II error of false nonpresence.

### 3.6 Habitat differences of salt marsh harvest mouse and California vole

*Reithrodontomys raviventris* and MICA generally spanned a similar range of site attributes (Figure S1.4). However, some differences in distributions were suggested by results of unpaired two‐sample *t* tests of covariate values at locations where RERA and MICA were both trapped (Table 8; Table S1.8). At sites where both species were trapped, values of ten covariates, denoting microsite conditions, differed significantly between the species. Attributes of trap sites with RERA captures differed from those with MICA captures in the following ways. RERA capture sites had significantly greater percent cover and a greater maximum height of the most dominant plant species (pickleweed). RERA sites also had a higher marsh elevation, higher MHW and MHHW levels (these two variables were significantly positively correlated; see Table S1.2), and higher elevation of trap location relative to MHW. RERA sites also had greater distance to agriculture, closer distance to nearest urban area, and greater marsh patch area not impended by barriers or channels >3m wide.

**Table 8:** Unpaired, two‐sample *t* tests with Bonferroni adjusted p‐values comparing trap site attributes for presence of salt marsh harvest mouse (RERA) and California vole (MICA)

Variable	*t*‐Value	*df*	*p*‐Value	MICA or RERA^a^
Marsh_Elev—marsh elevation measured from the trapping location extracted from DEMs, in cm	−5.236	1,165	<.001**	RERA
P1_V_PERC—percent cover of most dominant plant species	−3.224	1,298	.001**	RERA
P1_V1_MAX—maximum height of most dominant plant species, in cm	2.168	924	.030*	RERA
P1_V1_AVG—average height of the most dominant plant species, in cm	−1.117	921	.264	nd
MHW—mean high water level, in m	−5.152	783	<.001 **	RERA
Elev_MHW—elevation of trap location compared to mean high water, in m	−5.035	1,174	<.001**	RERA
MHHW—mean higher high water, in m	−5.152	783	<.001**	RERA
Elev_MHHW—mean higher high water	−5.018	1,174	<.001**	RERA
Dist_Levee—distance to closest levee, in m	−1.147	881	.252	nd
Dist_Water—distance to closest water, in m	−0.735	1,137	.462	nd
Dist_Bay—distance to bay, in m	1.437	1,316	.151	nd
Dist_Urban—distance to closest urban, in m	4.432	820	<.001**	MICA
Dist_Ag—distance to closest agriculture, in m	−4.039	816	<.001**	RERA
Dist_Road—distance to closest paved road, in m	−0.943	1,099	.346	nd
Patch_Size—size of patch of continuous marsh not impeded by barriers or channels >3 m wide or by levees, in ha	0.183	1,368	.854	nd
Patch_Size_Expanded—size of patch of continuous marsh not impeded by barriers or channels >3 m wide, in ha	−3.497	744	<.001**	RERA

a MICA or RERA = denotes which species had the higher mean value for the variable; nd = no significant difference in values between the two species.

* *p* < .05.

** *p* < .01.

## 4 DISCUSSION

Our study found that most of the RERA captures were associated with sites having pickleweed as the dominant ground‐cover category. Fewer RERA sites had bare ground as the dominant ground‐cover category, although about half of the RERA captures were associated with bare ground as the secondary ground‐cover category. RERA individuals are thought to disperse randomly during prebreeding, but are associated with pickleweed sites with midrange salinity levels during breeding and postbreeding (Padgett‐Flohr & Isakson, 2003). In Suisun Bay, RERA were associated with sites dominated with mixed vegetation or pickleweed (Sustaita, Quickert, Patterson, Barthman‐Thompson, & Estrella, 2011).

*Reithrodontomys raviventris* have long been associated with dense wetland cover with pickleweed as preferred habitat (Shellhammer, 1989; Shellhammer et al., 1982). Shellhammer et al. (1982) summarized trapping data from Suisun Bay and SFB, found that RERA was highly dependent on cover and was captured predominantly in areas of tall and dense pickleweed, did not detect RERA in pickleweed <6 in (15 cm) tall, and concluded that high tide refugia was an important feature for RERA habitat. Recent studies indicate that RERA are also present in significant numbers in brackish marshes and wetlands with managed hydrology (Smith, Riley, Barthman‐Thompson, Woo, et al., 2018; Sustaita et al., 2011). In Suisun Marsh, Sustaita et al. (2011) reported that higher RERA densities and reproductive potential, and postwinter persistence occurred in areas of pickleweed or in mixed wetland vegetation without pickleweed, whereas fewer RERAs were found in upland grassland.

Other studies of RERA have noted variable plant or habitat associations in San Pablo Bay, Suisun Marsh, and South SFB. At Mare Island by Vallejo, California, along San Pablo Bay, Bias and Morrison (1999) found that RERA mean home range was 2,133 m^2^, that RERA moved on average about 12 m per 2 hr, and that distances moved and home range sizes were largest in June and smallest in November. At the same study site, they later determined that plant species associations for RERA included denser areas of forbs, particularly fat hen or spear orach (*Atriplex patula*) being used more by males than females during summer through fall, and open areas of common pickleweed being used by both sexes at other times of the year (Bias & Morrison, 2006). In Suisun Bay, RERA feeding trials demonstrated dietary flexibility over season and habitat type where RERA consumed a variety of plants including rabbitsfoot grass (*Polypogon monspeliensis*), fat hen, pickleweed, watergrass (*Echinochloa crusgalli*), and alkali bulrush (*Bolboschoenus maritimus*; Smith & Kelt, 2019). Geissel, Shellhammer, and Harvey (1988) reported RERA use of more open areas with pickleweed in the presence of higher numbers of California voles (*M. californicus*), and RERA use of more closed areas with less pickleweed as numbers of voles declined. From a capture of 36 RERA in a managed marsh in Fremont, California, along South SFB, Basson (2009) reported finding the species to be randomly distributed with no association with pickleweed salinity, pickleweed height, distance to levees, distance to dry or filled water bodies, percent cover of vegetation, or sympatric rodents. Differential use of pickleweed sites by sex and season, as well as movement across various conditions (Geissel et al., 1988), might explain the findings by Basson (2009), as well as those by Botti, Warencyia, and Becker (1986) of several RERA in areas lacking pickleweed in the Suisun Marsh region of SFB.

We found that RERA presence correlates positively with marsh elevation, MHW level, and elevation of the trap location compared to MHW, particularly as compared with distributions of sympatric MICAs. This is consistent with other findings that RERA remained within the marsh in tall vegetation rather than swim to upland areas for high tide refugia sites (Smith, Barthman‐Thompson, Gould, & Mabry, 2014).

We found evidence of some degree of tolerance of RERA with levees and water (<200 m distance), and also with agricultural areas and paved roads (<2 km distance). This was consistent with Bias and Morrison's (1999) radio‐telemetry study of RERA at Mare Island, Solano County, California (Figure 2), who concluded that the species crossed roads, levees, and canals. However, RERA in our study did not apparently select for such conditions, as they were also associated with larger marsh patch sizes not interrupted by levees and roads.

We captured nine species of small mammals and found no specific evidence RERA being obligately associated with other small mammal species. Overall, the tidal marshes in the areas sampled have low small mammal detections where empty traps were encountered 73% of all trap nights. RERA captures constituted only 20% of all captures, compared with the more ubiquitous MICA (47%). In contrast, Bias and Morrison (2006) found RERA in 63% of all captures, with the next most frequently captured (29%) species being house mouse and with MICA constituting only 6% of all captures.

Few studies are available on RERA interactions with other small mammal species, such as with nonnative house mice or the occurrence of voles. Bias and Morrison (2006) found differences in habitat use between RERA and house mouse in tidal sites suggesting that house mice were using habitats that were more patchy or fragmented than habitats of RERA. Our current study found what seemed to be some differentiation of habitat and site attributes between RERA and MICAs, which might suggest some niche differentiation at the trap‐site scale, but we cannot definitively conclude that interaction (e.g., competition) between the species accounted for the differences. We did not detect evidence of either a positive or a negative relationship of RERA with other small mammal species, although it is possible that any competitive interactions may not have been detected because of the low numbers of trap captures of RERA and other small mammal species in North SFB.

We found that RERA presence, as compared with MICA presence, is characterized as being higher in marsh elevation, higher in MHW level, and higher in elevation compared with MHW. Also, as compared with MICA, RERA occurred in sites with greater height and percent cover of pickleweed, that were closer to urban areas but further from agricultural areas (these two variables were strongly negatively correlated; see Table S1.3), and that were larger in marsh patch size. Similarly, in a study of *Spartina* marshes along the southwest Atlantic coast of the United States, Canepuccia, Pascual, Biondi, and Iribarne (2015) found that occurrence of small mammals was related to vegetation cover and diversity, and that small mammal species composition varied by landscape context and configuration.

A Type II error may be more egregious if models predict RERA nonpresence than when this State‐ and Federally listed endangered species is actually present. That is, false absences (Type II error) of an endangered species have a greater potential negative consequence for conserving population viability than do false presences (Type I error). Predicting absence when the species is present could result in no specific habitat management being directed to such locations, potentially resulting habitat degradation or loss for the species. In such cases, models cannot substitute for on‐the‐ground surveys. This type of habitat association model is better suited to assess RERA presence than to conclude absence. We note, too, that we have not analyzed any temporal trends or differences in habitat associations over the course of the trapping seasons and years, assuming that the availability of environmental conditions in each trapping location remained constant over time. We also note that studies are lacking on identifying specific threats to population viability of the species, other than inferences made on threats of habitat changes, which aligns with the recommended research priorities to further RERA recovery, including range‐wide population estimates, demographics, and dynamics (Smith, Riley, Barthman‐Thompson, Statham, et al., 2018).

Additional threats to small mammals in tidal marshes and potentially to RERA conservation may yet appear or confound recovery, such as uptake of environmental toxins as documented by Clark, Foerster, Marn, and Hothem (1992). BN models for habitat associations can be informative for tidal marsh restorations that may be able to accommodate higher marsh elevations in relation to inundation and larger marsh patches in their designs. Ultimately, management and conservation of RERA, particularly under threats of climate change, sea level rise, and anthropogenic alteration of habitats, may need to take an ecosystem‐level approach (e.g., Stagg et al., 2016) that includes tracking increased inundation and flooding of pickleweed habitat from rising sea level (Field, Bayard, et al., 2016; Kirwan et al., 2010; Rosencranz et al., 2018).

The current study could not compare RERA occurrence with attributes of random‐site locations, so resource selection functions of the mouse for particular habitat attributes could not be determined. This could be a valuable topic for future studies, to better understand RERA habitat selection for guiding site management, especially considering landscape level changes associated with sea level rise and climate change. Further studies could also address existing or emerging threats to the species.

## CONFLICT OF INTEREST

The authors assert no conflict of interest with this research, manuscript, or related materials.

## AUTHOR CONTRIBUTIONS

B.G.M. devised and conducted the Bayesian network modeling analyses and conceived the manuscript; I.W. led the field trapping efforts and assisted with writing; K.M.T. conceived the manuscript, assisted in data summary, and assisted with writing; C.M.F. conducted data summary, geospatial analyses, and assisted with methods writing; G.R.G. provided logistic and administrative support, and assisted with writing.

## Data Availability

The database used for model creation, with metadata documentation, in spreadsheet (Microsoft Inc., Excel.xlsx) format, along with the Bayesian network models (Norsys Inc., Netica.dne format), are available in the Appendix S1.  Small mammal survey data and environmental covariates will be archived at the U.S. Geological SurveyScience Base Catalog (https://doi.org/10.5066/P96Q5D2T).
