# Supplementary material for: Habitat of the endangered salt marsh harvest mouse (Reithrodontomys raviventris) in San Francisco Bay
Source: Ecol Evol. 2020 Jan 7;10(2):662–77. doi: 10.1002/ece3.5860 (PMC6988558; doi:10.1002/ece3.5860)

Appendix S1

for:

Marcot, B. G., I. Woo, K. Thorne, C. Freeman, and G. R. Guntenspergen. Submitted. Habitat of the endangered salt marsh harvest mouse (*Reithrodontomys raviventris*) in San Francisco Bay. for: Diversity and Distributions

v. 12 July 2019

Table S1.1 Variables and modeling covariates used in the modeling of salt marsh harvest mouse habitat and environmental relationships. See text for methods and Metadata S1 for data.

| **Covariate set** \1 | **Variable name** | **Definition** |
| --- | --- | --- |
| C1. Trap Data | DATE ** | Date of the observed capture data in the form of (MM/DD/YYYY) |
|  | YEAR | Year of the observed capture data in the form of (YYYY) |
|  | Session ** | Unique number identifying a particular trapping session time period (not site specific, one trapping session could include multiple sites) |
|  | Unique_ID ** | Unique ID in the form of (YYYY_Site_Location_Session) |
|  | SITE | Site where trapping occurred (Tolay Creek, BM=Ben Mar marsh, GC= Guadal Canal, Fagan, Corte Madera, Tubbs Island Setback) |
|  | METHOD | Trapping method used (grid, transect, random) |
|  | LOCATION | Location where trapping occurred (NA=Random or Transect, Other identifiers are grid locations (i.e., T1) |
|  | Day | Specific date on which the trap was baited and open |
|  | TRAPNO ** | Unique identifier for individual taps |
|  | OBS ** | Observers conducting trapping and recording data |
| C2. Latitude - Longitude | Easting | X coordinate of the trapping location |
|  | Northing | Y coordinate of the trapping location |
| C3. Vegetation & Cover | P1_V1 | P1 = closest vegetation sampling plot to the trapping location  V1 = most dominant plant species in that plot |
|  | P1_V1_Perc | Percent cover of the most dominant plant species in P1 |
|  | P1_V1_Max | Maximum height of the most dominant plant species in P1, cm |
|  | P1_V1_Avg | Average height of the most dominant plant species in P1, cm |
| C4. Vegetation & Cover (also includes all C3 covariates, above) | P1_V2 | P1 = closest vegetation sampling plot to the trapping location  V2 = second most dominant plant species in that plot |
|  | P1_V2_Perc | Percent cover of the second most dominant plant species in P1 |
|  | P1_V2_Max | Maximum height of the second most dominant plant species in P1, cm |
|  | P1_V2_Avg | Average height of the second most dominant plant species in P1, cm |
|  | P1_V3 | P1 = closest vegetation sampling plot to the trapping location  V3 = third most dominant plant species in that plot |
|  | P1_V3_Perc | Percent cover of the third most dominant plant species in P1 |
|  | P1_V3_Max | Maximum height of the third most dominant plant species in P1, cm |
|  | P1_V3_Avg | Average height of the third most dominant plant species in P1, cm |
| C5. Elevation | Marsh_Elev | Marsh elevation of the trapping location, in m; extracted from digital elevation models (DEM) built from USGS RTK survey data or Lidar from the 2009-2011 CA Coastal Conservancy Coastal Lidar Project |
|  | MHW | Mean high water, in m; NAVD88 calculated from local tidal datums from climate change loggers for each specific site |
|  | Elev_MHW | Elevation of the trapping location compared to MHW (= Marsh_Elev - MHW), in m |
|  | MHHW * | Mean higher high water, in m; NAVD88 calculated from local tidal datums from climate change loggers for each specific site |
|  | Elev_MHHW | Elevation of the trapping location compared to MHHW (= Marsh_Elev - MHHW), in m |
| C6. Distance | Dist_Levee | Distance to closest levee, in m |
|  | Dist_Water | Distance to closest water, in m |
|  | Dist_Bay | Distance to Bay, in m |
|  | Dist_Urban * | Distance to closest urban, in m |
|  | Dist_Ag * | Distance to closest agriculture, in m |
|  | Dist_Road | Distance to closest road, in m, defined as paved road and not including levee or gravel roads |
| C7. Patch Size | Patch_Size | Continuous marsh not impeded by barriers of channels > 3m wide or levees, in ha |
|  | Patch_Size_Expanded | Continuous marsh not impeded by barriers of channels > 3m wide, in ha |

* Excluded from modeling due to high correlation, |r| ≥ 0.7 with other covariates; see Table S1.3.

** Covariate not used in modeling due to not being an environmental or response variable.
\1 Covariate sets as used in various Bayesian network model combinations; see Table S1.2.

Table S1.2. Plant species recorded in vegetation plots. Taxonomy as per Jepson Flora Project (2018). \1

| Family | Common name | Scientific name |
| --- | --- | --- |
| Chenopodiaceae | fat-hen | *Atriplex prostrata (*synonym *Atriplex triangularis)* |
| Chenopodiaceae | pickleweed | *Salicornia pacifica* (synonym *Sarcocornia* *pacifica*) |
| Asteraceae | brass-buttons | *Cotula coronopifolia* |
| Asteraceae | marsh gumplant | *Grindelia stricta var. angustifolia* |
| Asteraceae | marsh jaumea | *Jaumea carnosa* |
| Brassicaceae | black mustard | *Brassica nigra* |
| Brassicaceae | perennial pepperweed | *Lepidium latifolium* |
| Caryophyllaceae | sand spurrey | *Spergularia* spp. |
| Cyperaceae | saltmarsh bulrush | *Bolboschoenus* *maritimus subsp. paludosus* |
| Cyperaceae | Olney’s three-square bulrush | *Schoenoplectus americanus* |
| Fabaceae | sourclover | *Melilotus indicus* |
| Frankeniaceae | alkali heath | *Frankenia salina* |
| Plantaginaceae | plantain | *Plantago* sp. |
| Poaceae | salt grass | *Distichlis spicata* |
| Poaceae | meadow barley | *Hordeum brachyantherum* |
| Poaceae | rabbitfoot grass | *Polypogon monspeliensis* |
| Poaceae | California cord grass | *Spartina foliosa* |
| Polygonaceae | prostrate knotweed | *Polygonum aviculare subsp. depressum* |
| Rosaceae | Pacific silverweed | *Potentilla anserine subsp. pacifica* |
| Typhaceae | cattail | *Typha* sp., prob. *T. angustifolia*, or *T.* x *glauca* hybrid between *T. angustifolia* and *T. latifolia* |

\1 Jepson Flora Project (eds.) 2018. Jepson eFlora, <http://ucjeps.berkeley.edu/eflora/>, accessed August 07, 2018.

Table S1.3. Pearson correlations (r) among environmental and habitat covariates (see Appendix S1: Fig. S1 for definitions). High correlation values of |r| ≥ 0.7 are denoted in the cells below with the yellow background.

| Covariate | Marsh Elev | MHW | Elev MHW | MHHW | Elev MHHW | Dist Levee | Dist Water | Dist Bay | Dist Urban | Dist Ag | Dist Road | Patch Size | Patch Size Expanded |
| --- | --- | --- | --- | --- | --- | --- | --- | --- | --- | --- | --- | --- | --- |
| Marsh Elev | 1 |  |  |  |  |  |  |  |  |  |  |  |  |
| MHW | 0.276 | 1 |  |  |  |  |  |  |  |  |  |  |  |
| Elev MHW | 0.998 | 0.216 | 1 |  |  |  |  |  |  |  |  |  |  |
| MHHW * | 0.274 | 0.999 | 0.214 | 1 |  |  |  |  |  |  |  |  |  |
| Elev MHHW | 0.998 | 0.212 | 1 | 0.21 | 1 |  |  |  |  |  |  |  |  |
| Dist Levee | -0.324 | 0.252 | -0.346 | 0.257 | -0.347 | 1 |  |  |  |  |  |  |  |
| Dist Water | -0.26 | -0.256 | -0.247 | -0.254 | -0.246 | 0.011 | 1 |  |  |  |  |  |  |
| Dist Bay | 0.068 | 0.174 | 0.058 | 0.176 | 0.057 | 0.383 | -0.049 | 1 |  |  |  |  |  |
| Dist Urban * | -0.315 | -0.974 | -0.257 | -0.968 | -0.253 | -0.175 | 0.262 | -0.281 | 1 |  |  |  |  |
| Dist Ag * | 0.12 | 0.799 | 0.071 | 0.784 | 0.068 | -0.049 | -0.134 | -0.11 | -0.844 | 1 |  |  |  |
| Dist Road | 0.116 | -0.612 | 0.157 | -0.608 | 0.16 | -0.162 | -0.282 | -0.486 | 0.688 | -0.61 | 1 |  |  |
| Patch Size | 0.035 | 0.048 | 0.033 | 0.051 | 0.032 | 0.29 | 0.415 | 0.047 | 0.002 | -0.06 | -0.08 | 1 |  |
| Patch Size Expanded | 0.343 | 0.623 | 0.308 | 0.633 | 0.305 | -0.204 | -0.12 | -0.049 | -0.544 | 0.318 | -0.33 | -0.02 | 1 |

* Covariates eliminated from subsequent modeling because of significant correlations.

Table S1.4. Results of testing Bayesian network models 1-19, predicting presence (RERA) and absence (NOTRERA) of salt marsh harvest mouse, against known trap outcomes. Listed are numbers of trap-night outcomes; total number of cases for each model = 3339.

| Model no. | (a) Predicted NOTRERA, actual NOTRERA | (b) Type I error: predicted RERA, actual NOTRERA | (c) Type II error: predicted NOTRERA, actual RERA | (d) Predicted RERA, actual RERA |
| --- | --- | --- | --- | --- |
| 1 | 2652 | 18 | 604 | 65 |
| 2 | 2670 | 0 | 669 | 0 |
| 3 | 2668 | 2 | 669 | 0 |
| 4 | 1583 | 1087 | 384 | 285 |
| 5 | 2670 | 0 | 669 | 0 |
| 6 | 2670 | 0 | 669 | 0 |
| 7 | 2670 | 0 | 669 | 0 |
| 8 | 2494 | 176 | 528 | 141 |
| 9 | 2494 | 176 | 528 | 141 |
| 10 | 2521 | 149 | 550 | 119 |
| 11 | 2474 | 196 | 522 | 147 |
| 12 | 2494 | 176 | 528 | 141 |
| 13 | 2521 | 149 | 550 | 119 |
| 14 | 2474 | 196 | 522 | 147 |
| 15 | 2648 | 22 | 626 | 43 |
| 16 | 2646 | 24 | 620 | 49 |
| 17 | 2648 | 22 | 626 | 43 |
| 18 | 2648 | 22 | 628 | 41 |
| 19 | 1333 | 1337 | 238 | 431 |

*Type I error = b / (b+d)*

*Type II error = c / (a+c)*

Table S1.5. Results of testing Bayesian network models 20-38, predicting presence of all 9 small mammal species (see text), against known trap outcomes. Listed are numbers of trap-night outcomes; total number of cases for each model = 3339. Type I and II error rates, across all models, for RERA were calculated from this breakdown of model performance.

| Model no. | (a) Predicted NOTRERA, actual NOTRERA | (b) Type I error: predicted RERA, actual NOTRERA | (c) Type II error: predicted NOTRERA, actual RERA | | | | (d) Predicted RERA, actual RERA | | (h) RERA only total prediction error \1 |
| --- | --- | --- | --- | --- | --- | --- | --- | --- | --- |
| 20 | 2596 | 74 | 552 | | | | 117 | | 19% |
| 21 | 2643 | 27 | 647 | | | | 22 | | 20% |
| 22 | 2670 | 0 | 669 | | | | 0 | | 20% |
| 23 | 2224 | 446 | 557 | | | | 112 | | 30% |
| 24 | 2655 | 15 | 654 | | | | 15 | | 20% |
| 25 | 2670 | 0 | 669 | | | | 0 | | 20% |
| 26 | 2670 | 0 | 669 | | | | 0 | | 20% |
| 27 | 2521 | 149 | 550 | | | | 119 | | 21% |
| 28 | 2643 | 27 | 647 | | | | 22 | | 20% |
| 29 | 2670 | 0 | 669 | | | | 0 | | 20% |
| 30 | 2486 | 184 | 529 | | | | 140 | | 21% |
| 31 | 2494 | 176 | 528 | | | | 141 | | 21% |
| 32 | 2471 | 199 | 525 | | | | 144 | | 22% |
| 33 | 2506 | 164 | 535 | | | | 134 | | 21% |
| 34 | 2636 | 34 | 606 | | | | 63 | | 19% |
| 35 | 2632 | 38 | 602 | | | | 67 | | 19% |
| 36 | 2638 | 32 | 606 | | | | 63 | | 19% |
| 37 | 2636 | 34 | 604 | | | | 65 | | 19% |
| 38 | 1526 | 1144 | 277 | | | | 392 | | 43% |
| COLOR KEY: |  |  |  |  |  |  |  |  |  |
| ≥ 20% | | | |  |  |  |  |  |  |
| < 20% | | | |  |  |  |  |  |  |

*Type I error = b / (b+d)*

*Type II error = c / (a+c)*

\1 Calculated as Type I error (b) + Type II error (c) / 3339 total cases.

Table S1.6. Results of 4-fold cross-validation of selected best Bayesian network models of salt marsh harvest mouse. Error rates were predicted by using the case file of trap results with the expectation maximization algorithm in the Bayesian network modeling program Netica (Norsys, Inc.); see text for explanation. RERA = salt marsh harvest mouse presence, NOTRERA = salt marsh harvest mouse absence.

|  |  | No. of cases | | | | Validation errors, RERA only | | |  |
| --- | --- | --- | --- | --- | --- | --- | --- | --- | --- |
| Model number | Fold | (a) Predicted NOTRERA, actual NOTRERA | (b) Type I error: predicted RERA, actual NOTRERA \1 | (c) Type II error: predicted NOTRERA, actual RERA  \2 | (d) Predicted RERA, actual RERA | Type I Error | Type II Error | Total Error |  |
| ***RERA Presence-Absence*** | | |  |  |  |  |  |  |  |
| Model 2 | Fold 1 | 680 | 0 | 155 | 0 | 0.0% | 18.6% | 18.6% |  |
|  | Fold 2 | 667 | 0 | 168 | 0 | 0.0% | 20.1% | 20.1% |  |
|  | Fold 3 | 663 | 0 | 172 | 0 | 0.0% | 20.6% | 20.6% |  |
|  | Fold 4 | 660 | 0 | 174 | 0 | 0.0% | 20.9% | 20.9% |  |
|  | **MEANS** | **667.5** | **0** | **167.25** | **0** | **0.0%** | **20.0%** | **20.0%** |  |
| Model 6 | Fold 1 | 680 | 0 | 155 | 0 | 0.0% | 18.6% | 18.6% |  |
|  | Fold 2 | 667 | 0 | 168 | 0 | 0.0% | 20.1% | 20.1% |  |
|  | Fold 3 | 663 | 0 | 172 | 0 | 0.0% | 20.6% | 20.6% |  |
|  | Fold 4 | 660 | 0 | 174 | 0 | 0.0% | 20.9% | 20.9% |  |
|  | **MEANS** | **667.5** | **0** | **167.25** | **0** | **0.0%** | **20.0%** | **20.0%** |  |
| Model 7 | Fold 1 | 680 | 0 | 155 | 0 | 0.0% | 18.6% | 18.6% |  |
|  | Fold 2 | 667 | 0 | 168 | 0 | 0.0% | 20.1% | 20.1% |  |
|  | Fold 3 | 663 | 0 | 172 | 0 | 0.0% | 20.6% | 20.6% |  |
|  | Fold 4 | 660 | 0 | 174 | 0 | 0.0% | 20.9% | 20.9% |  |
|  | **MEANS** | **667.5** | **0** | **167.25** | **0** | **0.0%** | **20.0%** | **20.0%** |  |
| ***All small mammal spp*** | | |  |  |  |  |  |  | All-spp. error \3 |
| Model 25 | Fold 1 | 3184 | 0 | 155 | 0 | 0.0% | 4.6% | 4.6% | 48.5% |
|  | Fold 2 | 3171 | 0 | 168 | 0 | 0.0% | 5.0% | 5.0% | 49.7% |
|  | Fold 3 | 3167 | 0 | 172 | 0 | 0.0% | 5.2% | 5.2% | 51.4% |
|  | Fold 4 | 3165 | 0 | 174 | 0 | 0.0% | 5.2% | 5.2% | 47.2% |
|  | **MEANS** | **3171.75** | **0** | **167.25** | **0** | **0.0%** | **5.0%** | **5.0%** | 49.2% |
| Model 26 | Fold 1 | 3184 | 0 | 155 | 0 | 0.0% | 4.6% | 4.6% | 50.0% |
|  | Fold 2 | 3171 | 0 | 168 | 0 | 0.0% | 5.0% | 5.0% | 48.6% |
|  | Fold 3 | 3167 | 0 | 172 | 0 | 0.0% | 5.2% | 5.2% | 51.0% |
|  | Fold 4 | 3165 | 0 | 174 | 0 | 0.0% | 5.2% | 5.2% | 47.2% |
|  | **MEANS** | **3171.75** | **0** | **167.25** | **0** | **0.0%** | **5.0%** | **5.0%** | 49.2% |

\1 Type I error = b / (b+d)

\2 Type II error = c / (a+c)

\3 Across prediction of presence/absence of all 9 small mammal species captured. Compare this column to the calibration "overall confusion error" in text Table 7.

Table S1.7. Sensitivity analyses of the best-fit Bayesian network models of salt marsh harvest mouse (RERA) presence-absence and all-species presence. Sensitivity was measured as entropy reduction (see text); higher values suggest greater sensitivity of the outcome variable to the given covariate.

| **Model** | **Covariate** | **Entropy reduction** |
| --- | --- | --- |
| *RERA presence-absence models* | |  |
| Model 2 | Easting | 0.015 |
|  | Northing | 0.004 |
| Model 6 | DistRoad | 0.007 |
| Model 7 | PatchSize | 0.018 |
|  | PatchSizeExp | 0.012 |
| *All-species presence models* | |  |
| Model 25 | DistRoad | 0.025 |
| Model 26 | PatchSize | 0.219 |
|  | PatchSizeExp | 0.059 |

Table S1.8. Calibration accuracy (classification confusion error) rates of Bayesian network models predicting presence of salt marsh harvest mouse (RERA) and California vole (MICA).

|  | Confusion table outcomes, for calculating RERA-MICA overlap errors (no. of cases) | | | | | Error rate (%), RERA-MICA overlap errors | | |
| --- | --- | --- | --- | --- | --- | --- | --- | --- |
| Model no. | (a) predicted MICA, actual MICA | (b) predicted MICA, actual RERA | (c) predicted RERA, actual RERA | (d) predicted RERA, actual MICA | (e)  Total no. cases | (f) correct predictions for MICA and RERA | (g) predicted MICA, actual RERA | (h) predicted RERA, actual MICA |
| 20 | 1408 | 449 | 117 | 58 | 2032 | 75% | 22% | 3% |
| 21 | 1422 | 561 | 22 | 16 | 2021 | 71% | 28% | 1% |
| 22 | 1448 | 571 | 0 | 0 | 2019 | 72% | 28% | 0% |
| 23 | 1158 | 454 | 112 | 272 | 1996 | 64% | 23% | 14% |
| 24 | 1437 | 546 | 15 | 10 | 2008 | 72% | 27% | 0% |
| 25 | 1557 | 649 | 0 | 0 | 2206 | 71% | 29% | 0% |
| 26 | 1185 | 501 | 0 | 0 | 1686 | 70% | 30% | 0% |
| 27 | 1218 | 410 | 119 | 130 | 1877 | 71% | 22% | 7% |
| 28 | 1348 | 529 | 22 | 16 | 1915 | 72% | 28% | 1% |
| 29 | 1428 | 551 | 0 | 0 | 1979 | 72% | 28% | 0% |
| 30 | 1206 | 404 | 140 | 152 | 1902 | 71% | 21% | 8% |
| 31 | 1218 | 410 | 141 | 146 | 1915 | 71% | 21% | 8% |
| 32 | 1230 | 410 | 144 | 146 | 1930 | 71% | 21% | 8% |
| 33 | 1230 | 410 | 134 | 140 | 1914 | 71% | 21% | 7% |
| 34 | 1457 | 501 | 63 | 22 | 2043 | 74% | 25% | 1% |
| 35 | 1445 | 494 | 67 | 24 | 2030 | 74% | 24% | 1% |
| 36 | 1460 | 500 | 63 | 22 | 2045 | 74% | 24% | 1% |
| 37 | 1454 | 496 | 65 | 22 | 2037 | 75% | 24% | 1% |
| 38 | 507 | 149 | 392 | 134 | 1182 | 76% | 13% | 11% |
|  |  |  |  |  |  | 72% | 24% | 4% |

\--------- means ---------/

| COLOR KEY: |  |
| --- | --- |
|  | error rate > 20% |
|  | error rate ≤ 20% |
|  | error rate ≤ 10% |

| RERA = salt marsh harvest mouse, *Reithrodontomys raviventris* |
| --- |
| MICA = California vole, *Microtus californicus* |

Figure S1.1. Marsh elevation metrics by study area site, box plots showing variation among trap locations for each site. See NOAA glossary (<https://shoreline.noaa.gov/glossary.html>) for definitions.


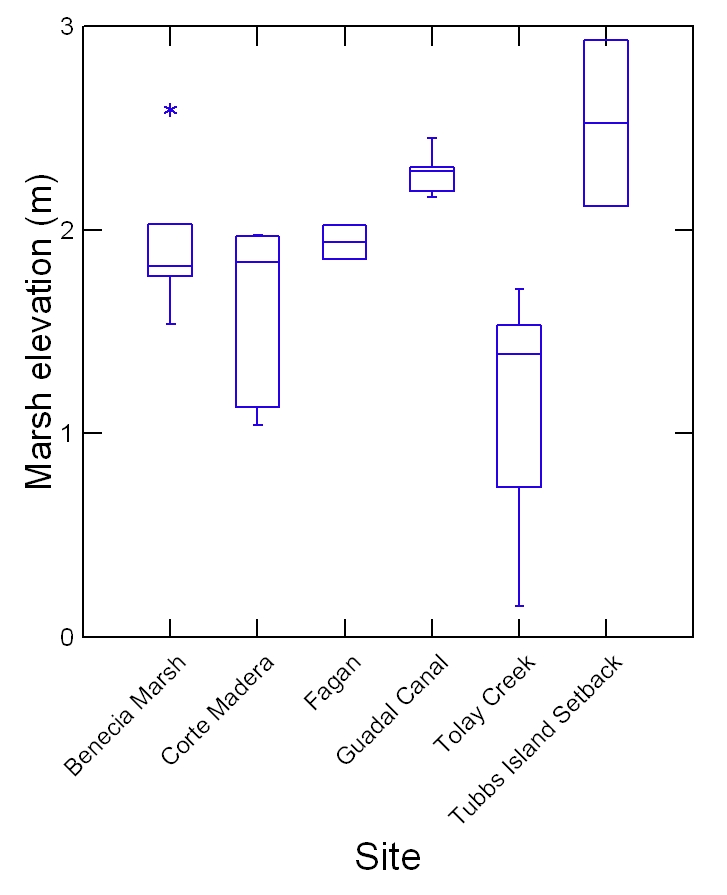


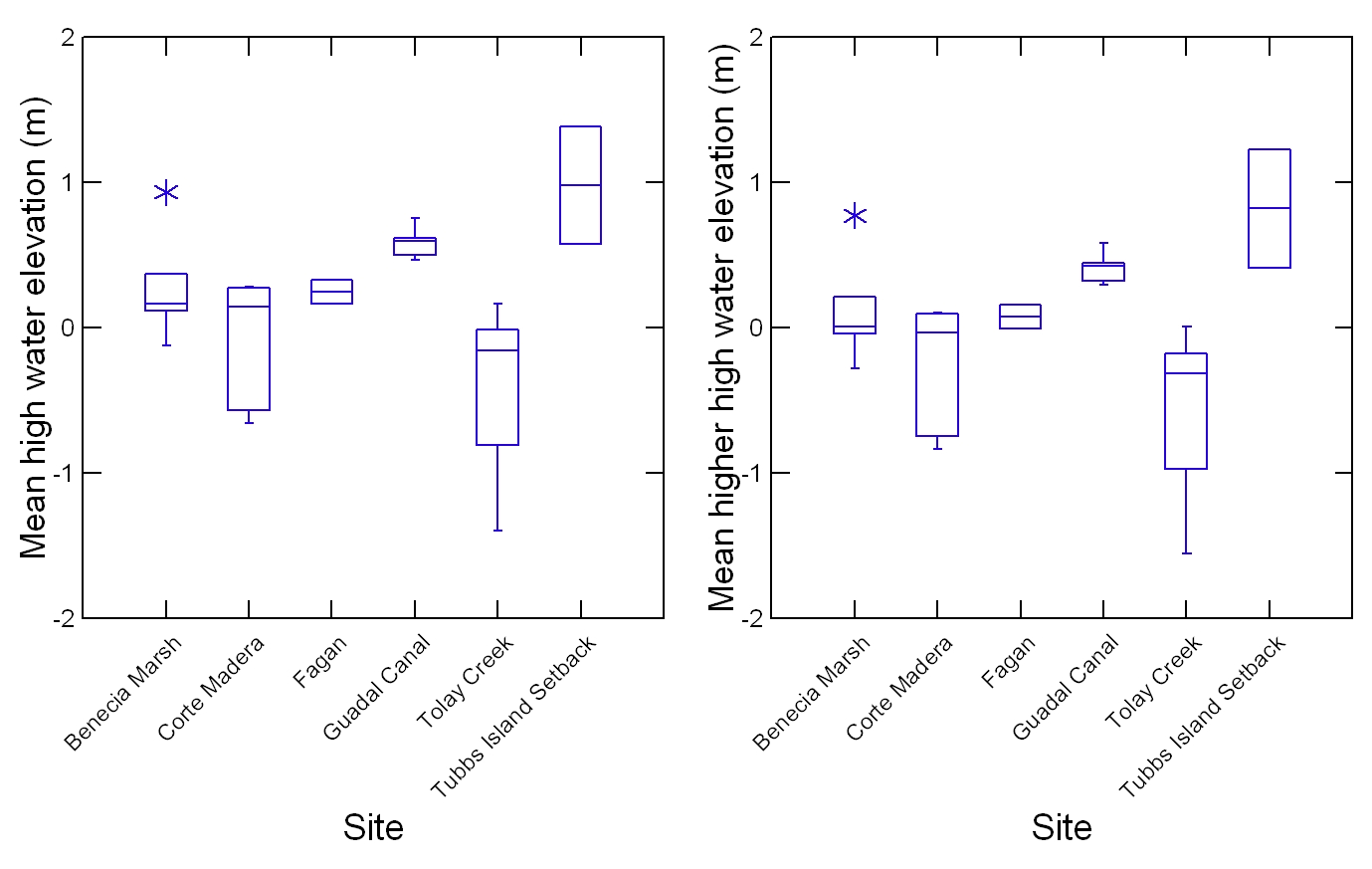


Figure S1.2. Distance of each trap site to nearest anthropogenic feature by study area site, box plots showing variation among trap locations for each site.


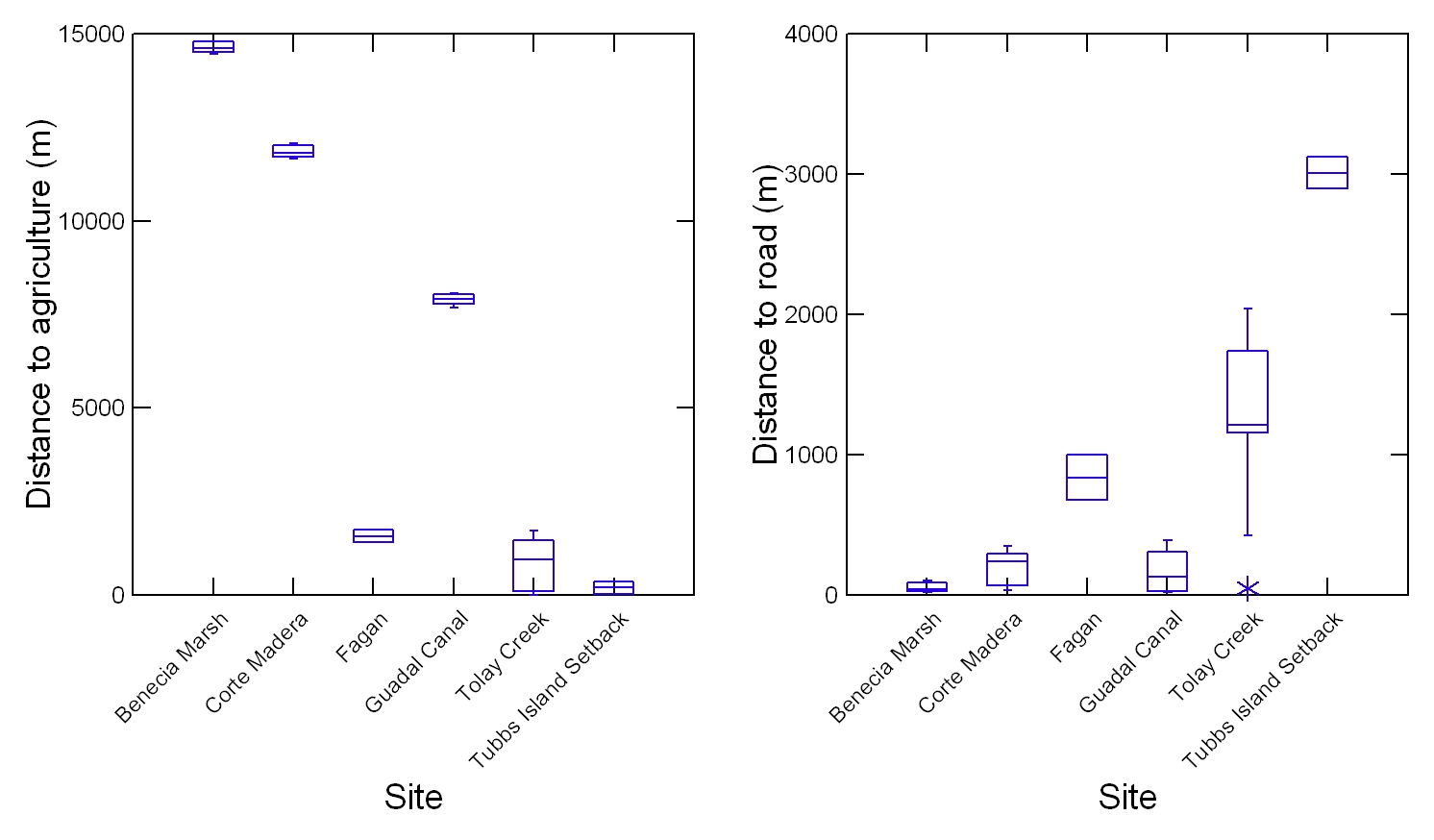


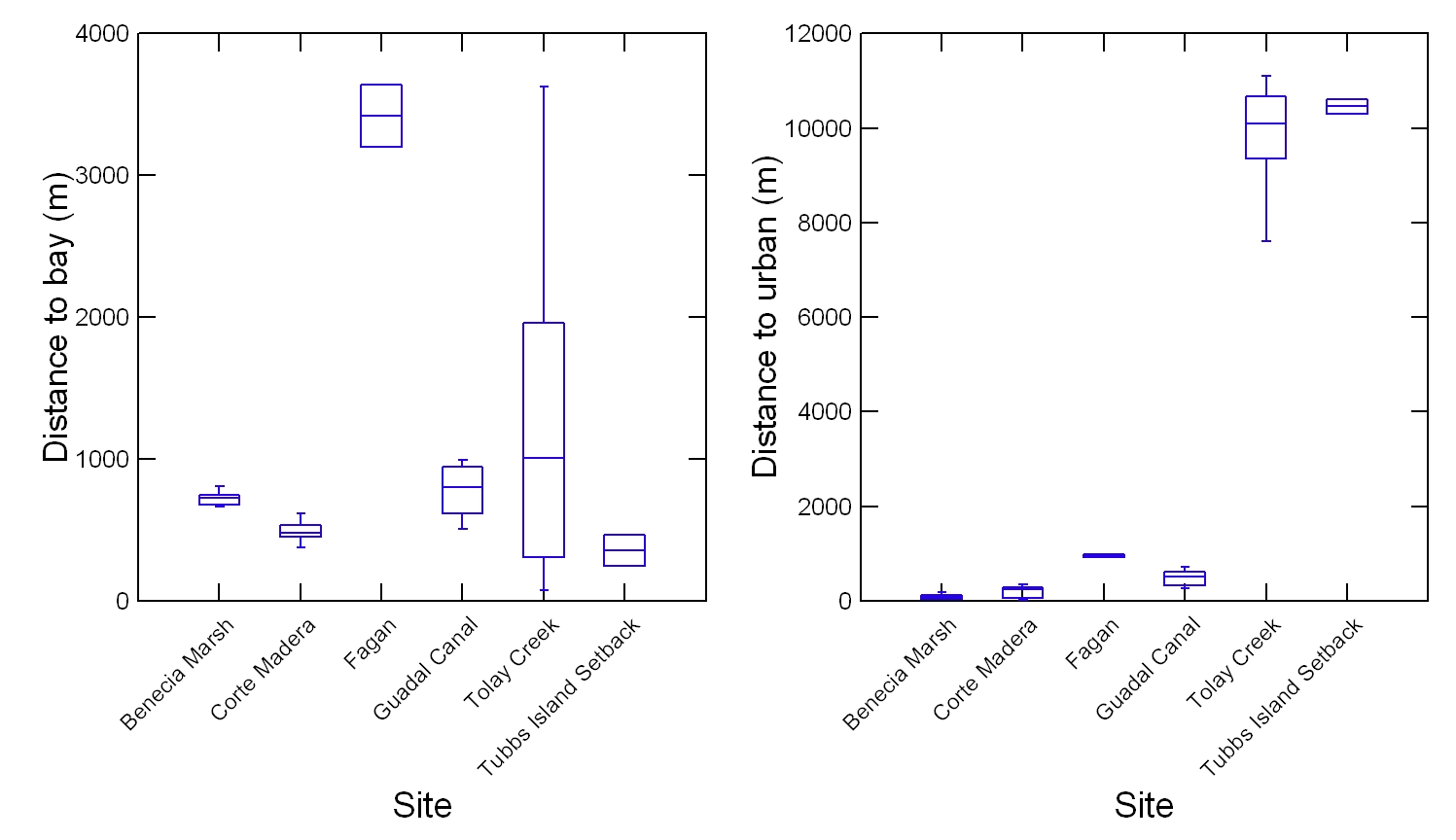


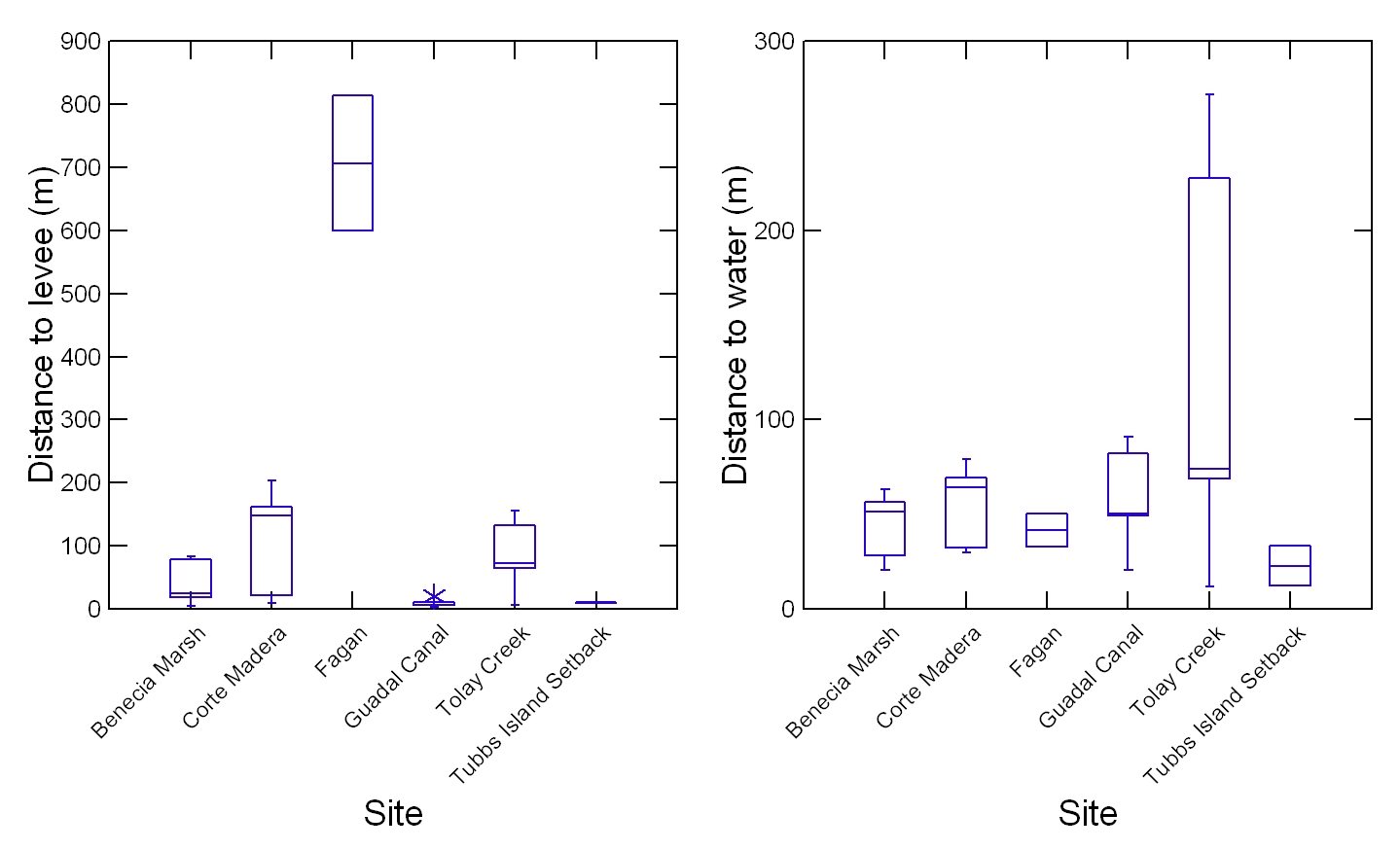


Figure S1.3. Habitat patch size metrics by study area site, box plots showing variation among trap locations for each site.


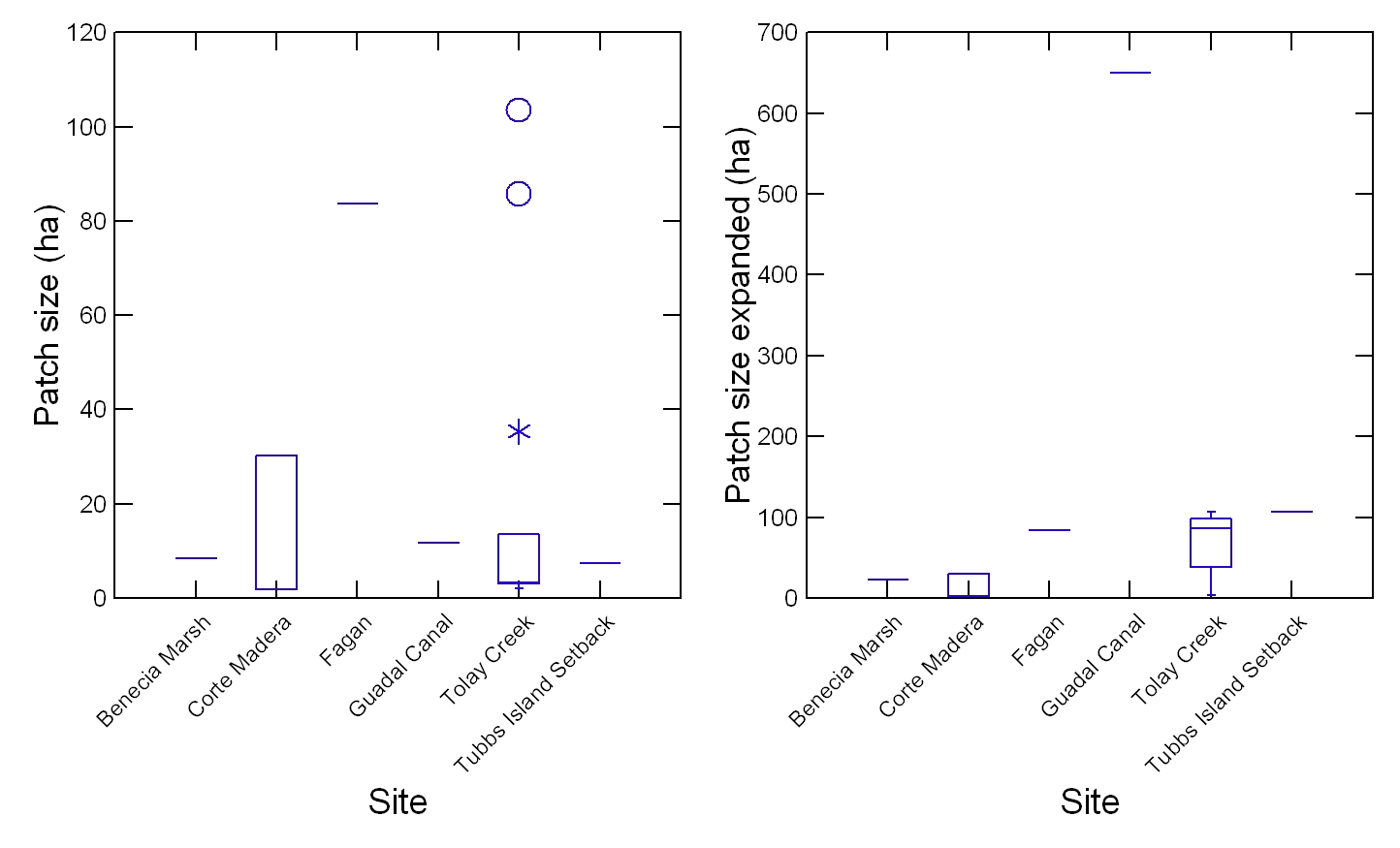


Figure S1.4. Box-plot comparisons of site attributes with captures of California vole (*Microtus californicus*, MICA) and salt marsh harvest mouse (*Reithrodontomys raviventris*, RERA), across all sites. See Appendix S1 Table S1 for variable name definitions.

Longitude (easting) and latitude (northing):


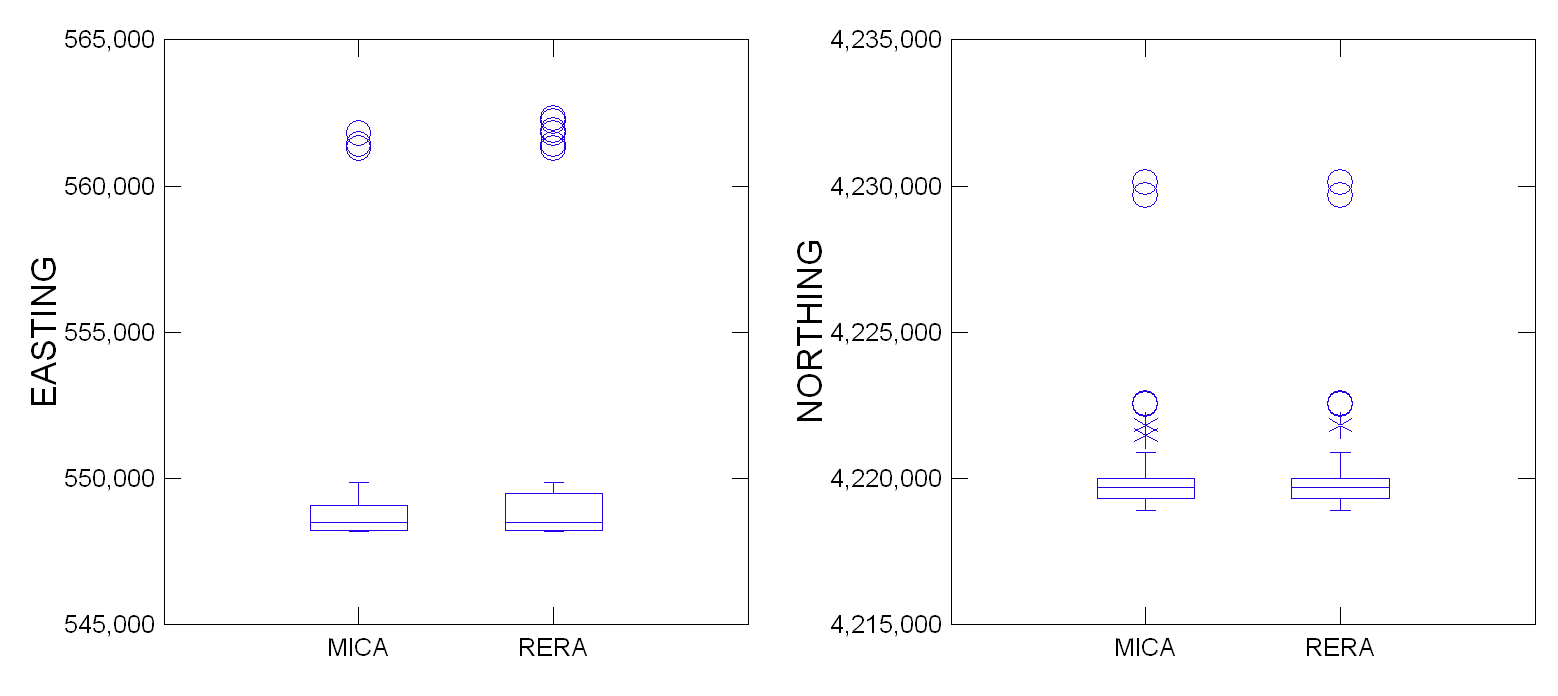


Marsh and water elevations:


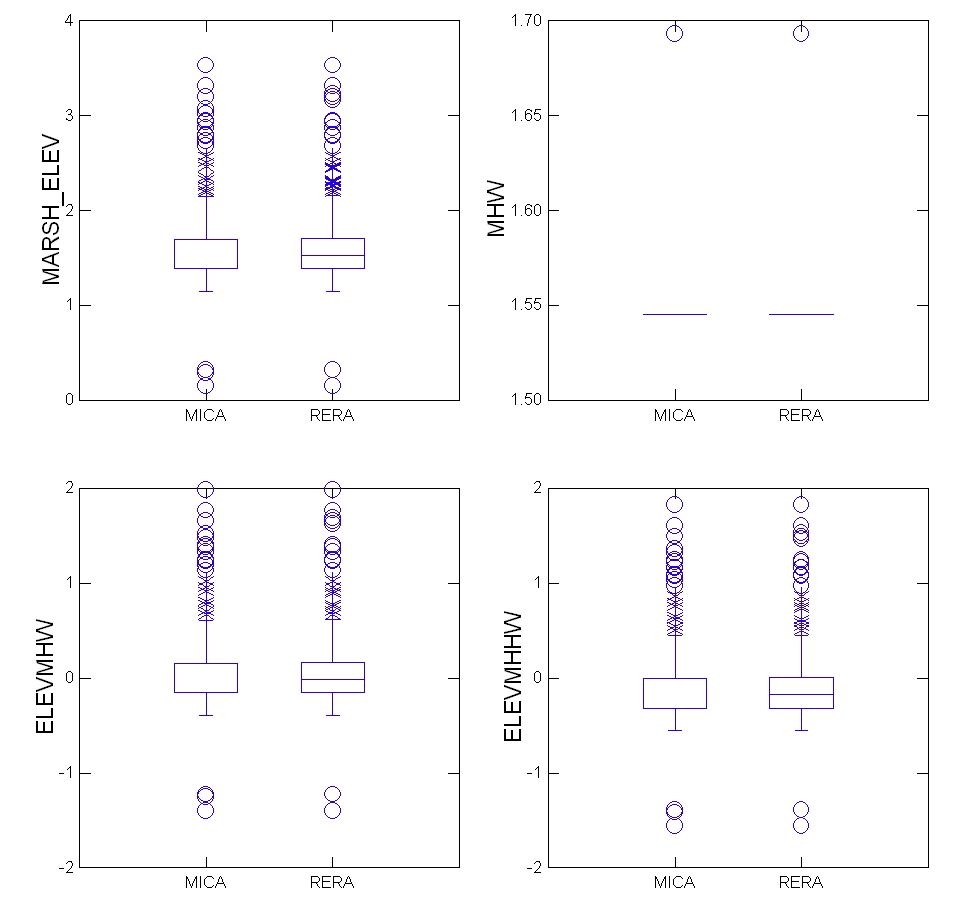


Distance to natural or anthropogenic features:


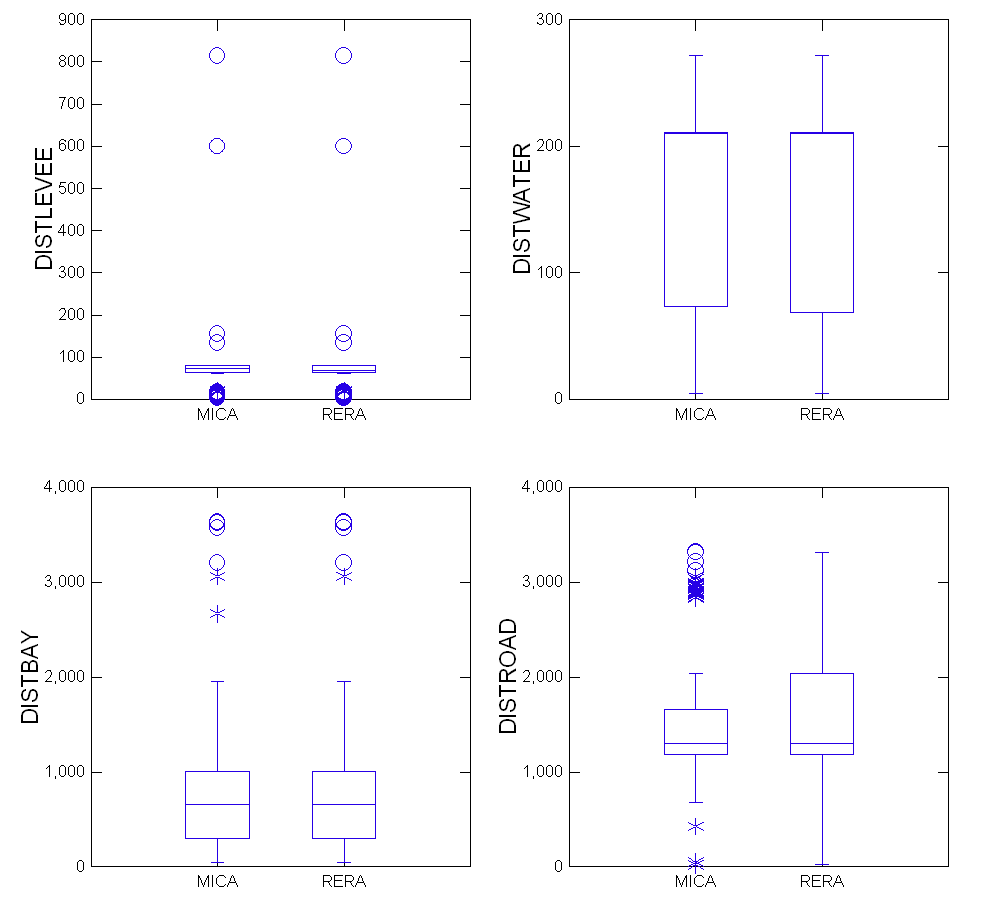


Marsh patch size:


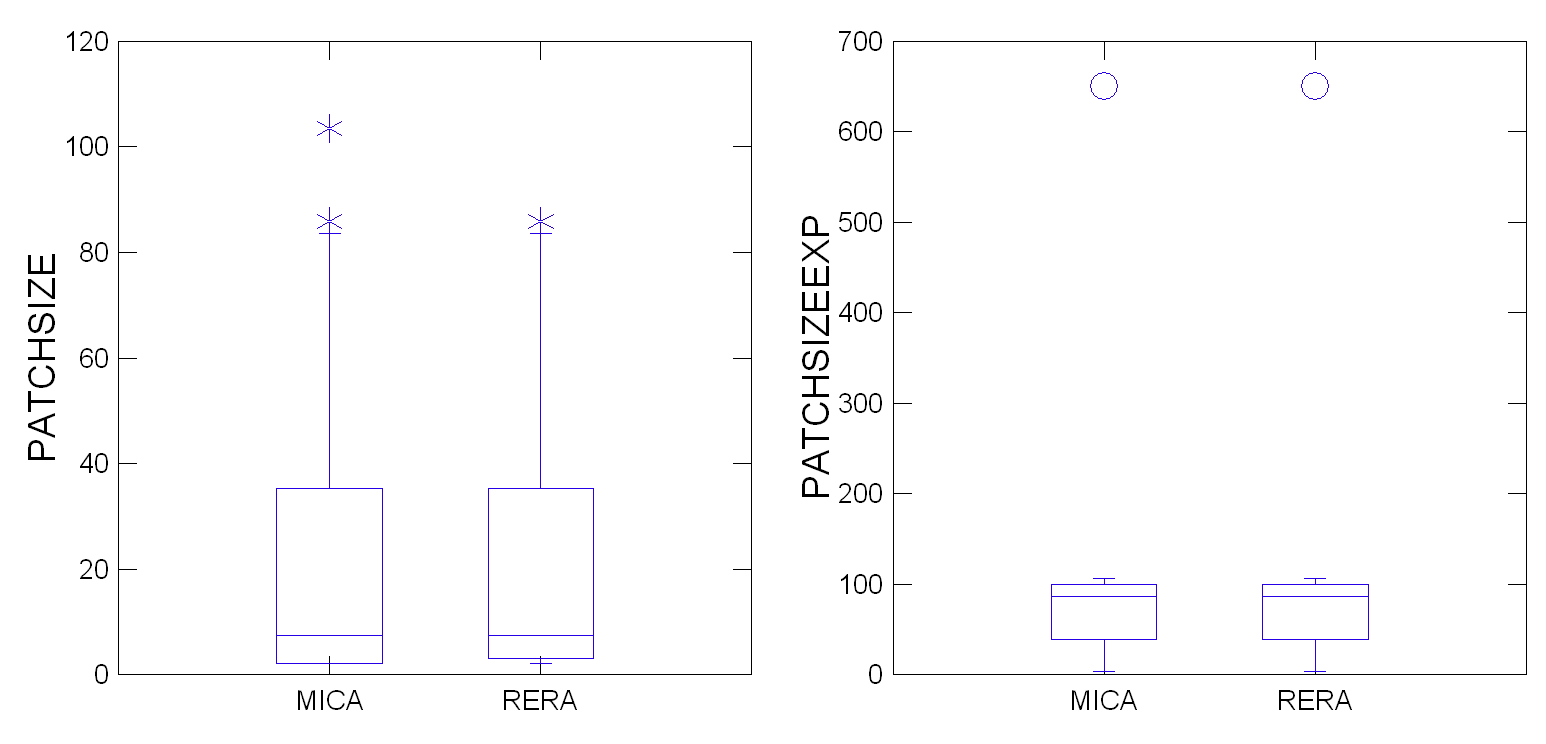


Figure S1.5. Bayesian network model configurations

The following figures display the specific configuration of each naïve Bayesian network model developed in the software Netica, as explained in text section 5 above. All Netica computer files are available. Note that all model structures were derived from empirical associations from field data, so that linkage among variables may variously represent ecological causal or noncausal associational relationships. The naïve Bayesian network is structured with links extending from the response variable to covariates, representing the degree to which variation in the response variable is explained by the covariates.

MODEL SET 1: SALT MARSH HARVEST MOUSE (RERA) PRESENCE-ABSENCE

Model no.: 1

Netica model file name: "RERA P-A Model 01.neta"


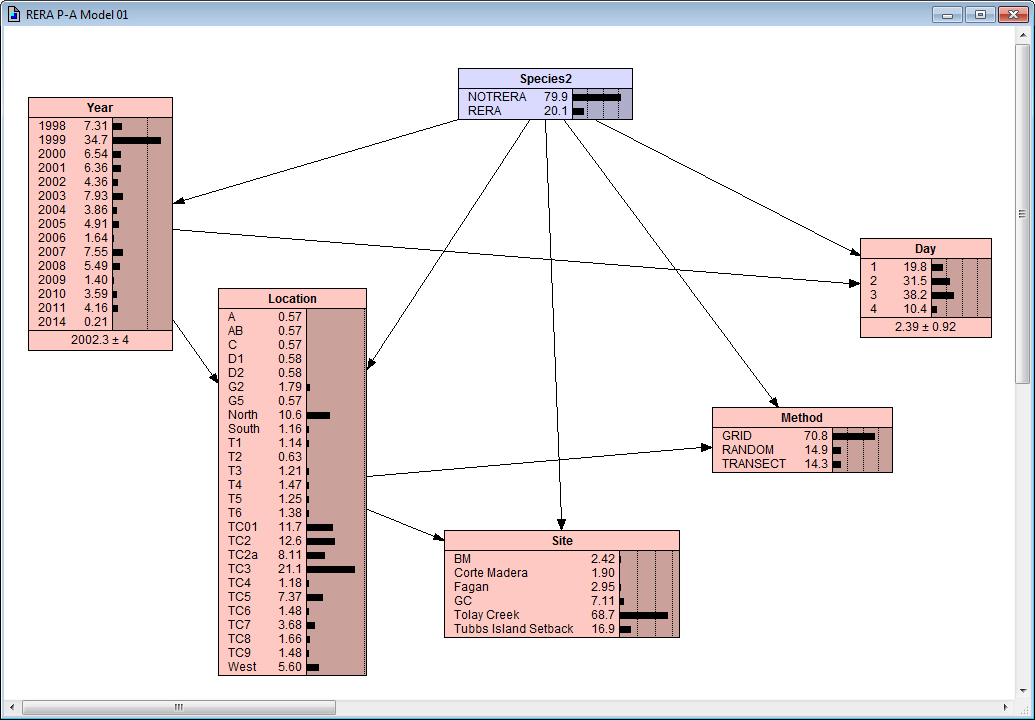


Model no.: 2

Netica model file name: "RERA P-A Model 02.neta"


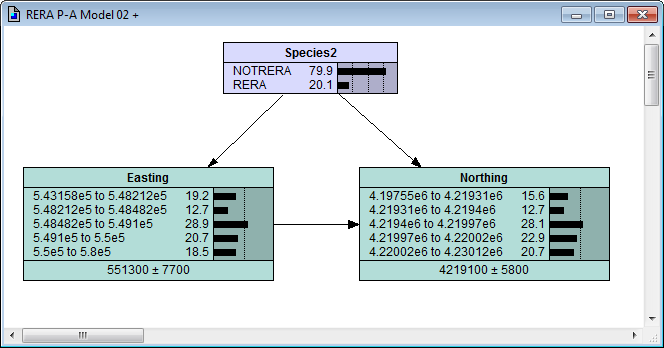


Model no.: 3

Netica model file name: "RERA P-A Model 03.neta"


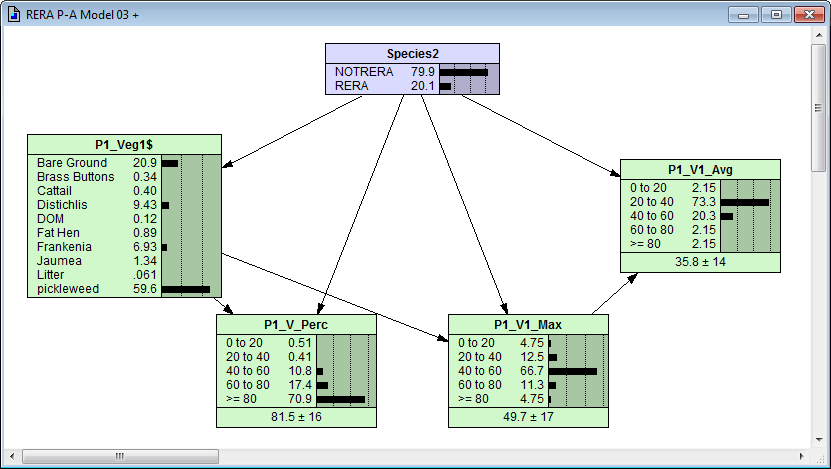


Model no.: 4

Netica model file name: "RERA P-A Model 04.neta"


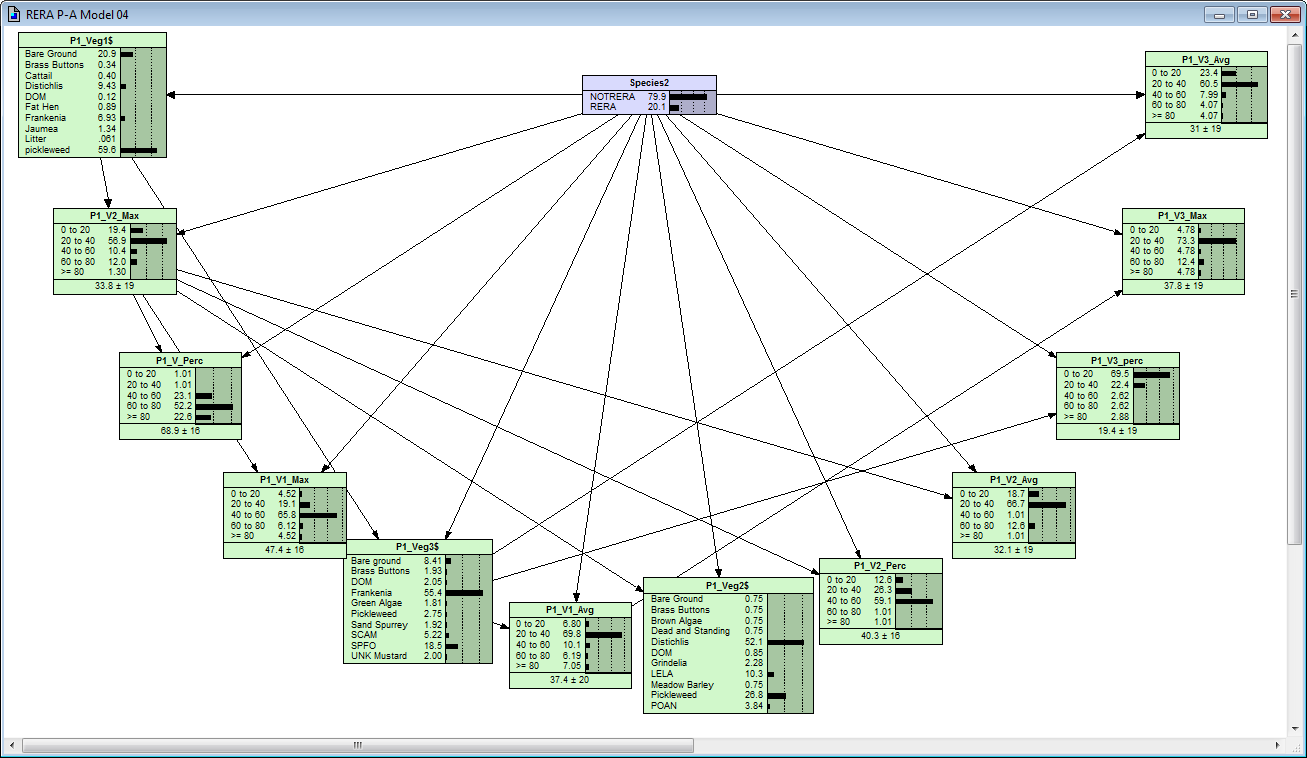


Model no.: 5

Netica model file name: "RERA P-A Model 05.neta"


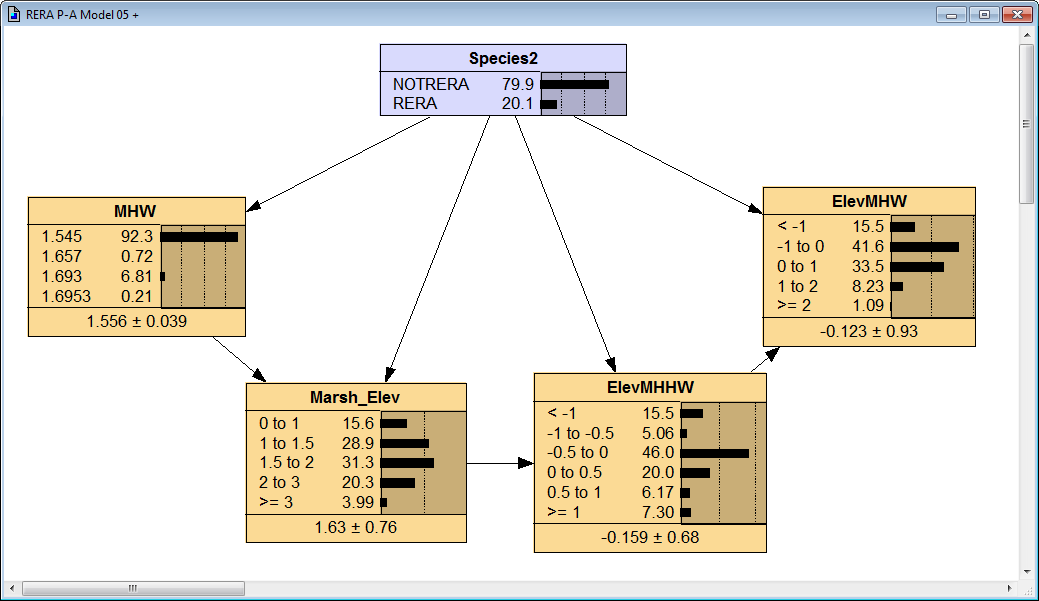


Model no.: 6

Netica model file name: "RERA P-A Model 06.neta"


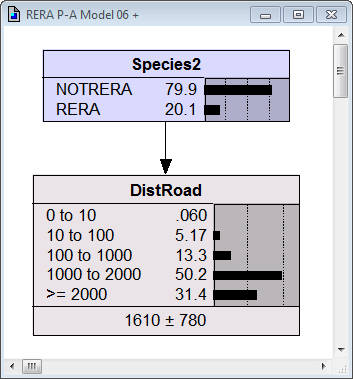


Model no.: 7

Netica model file name: "RERA P-A Model 07.neta"


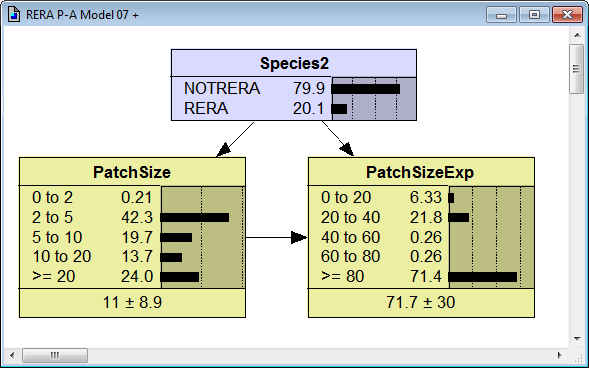


Model no.: 8

Netica model file name: "RERA P-A Model 08.neta"


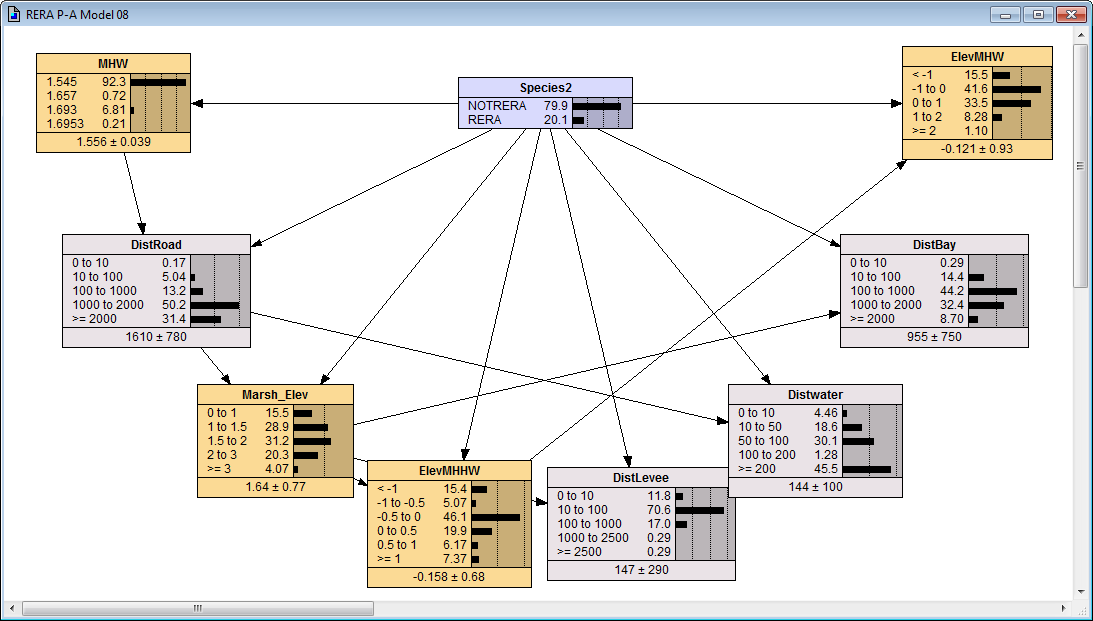


Model no.: 9

Netica model file name: "RERA P-A Model 09.neta"


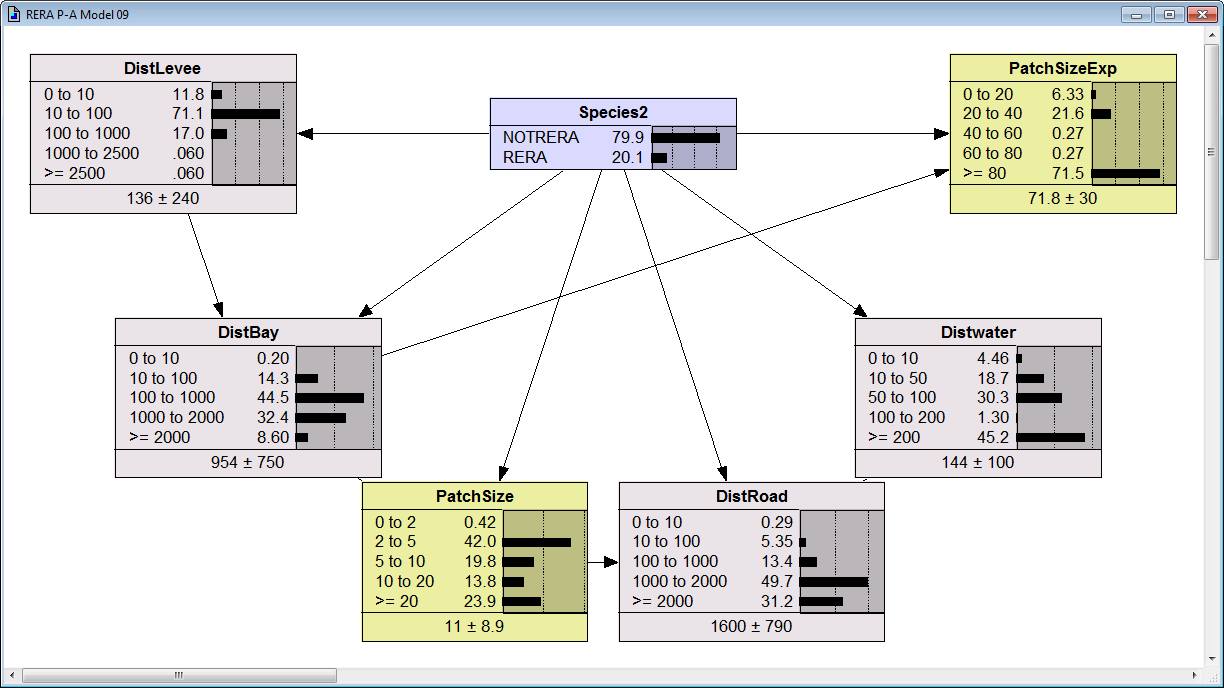


Model no.: 10

Netica model file name: "RERA P-A Model 10.neta"


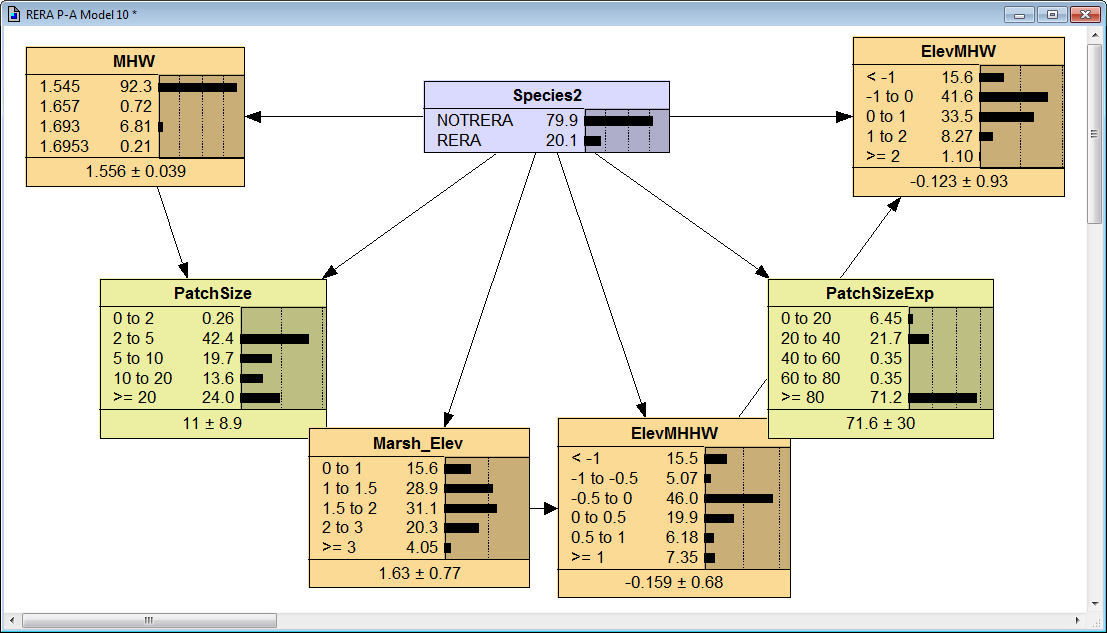


Model no.: 11

Netica model file name: "RERA P-A Model 11.neta"


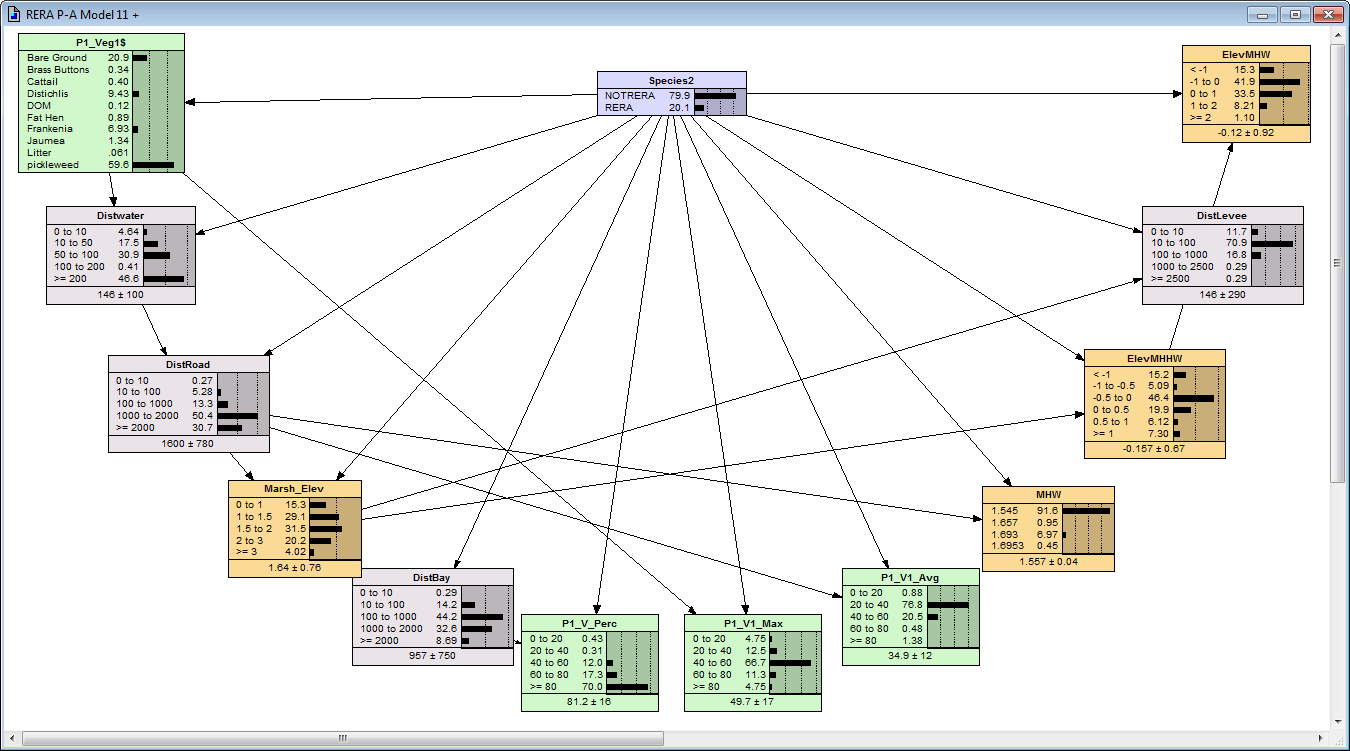


Model no.: 12

Netica model file name: "RERA P-A Model 12.neta"


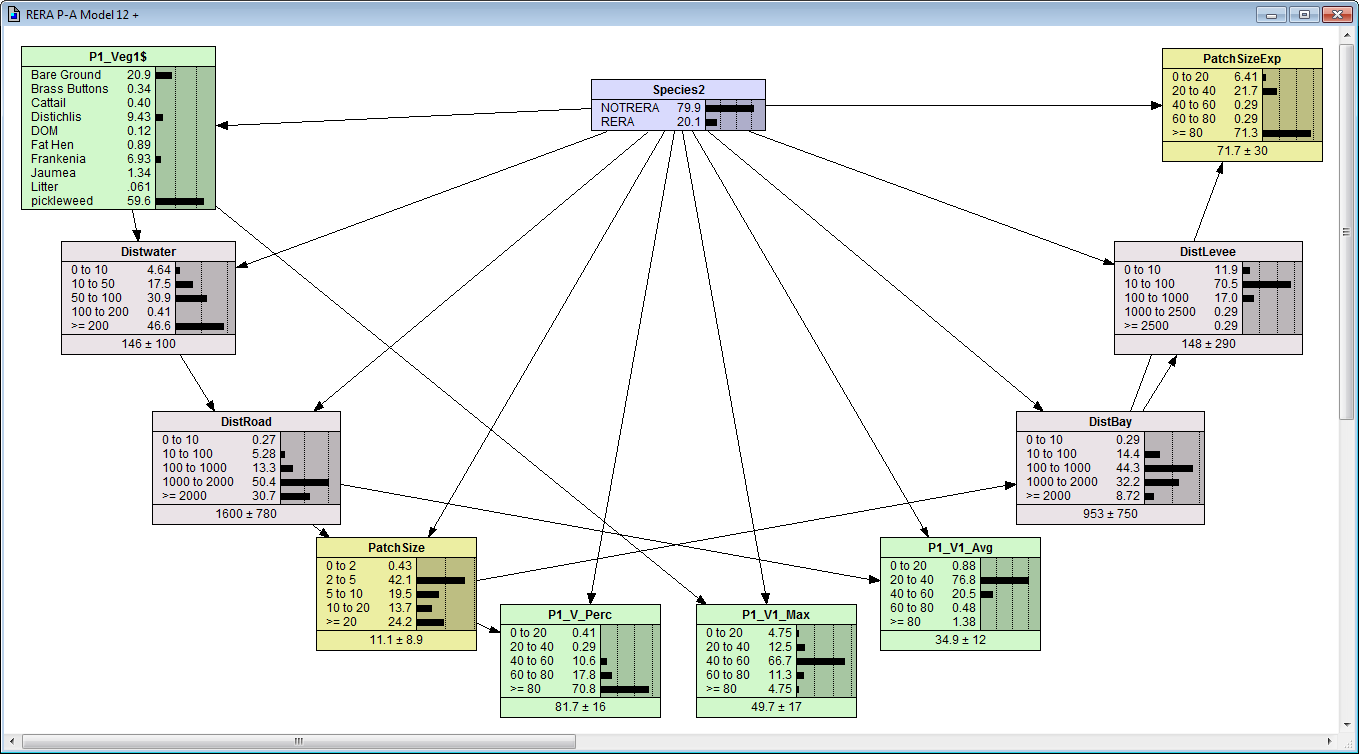


Model no.: 13

Netica model file name: "RERA P-A Model 13.neta"


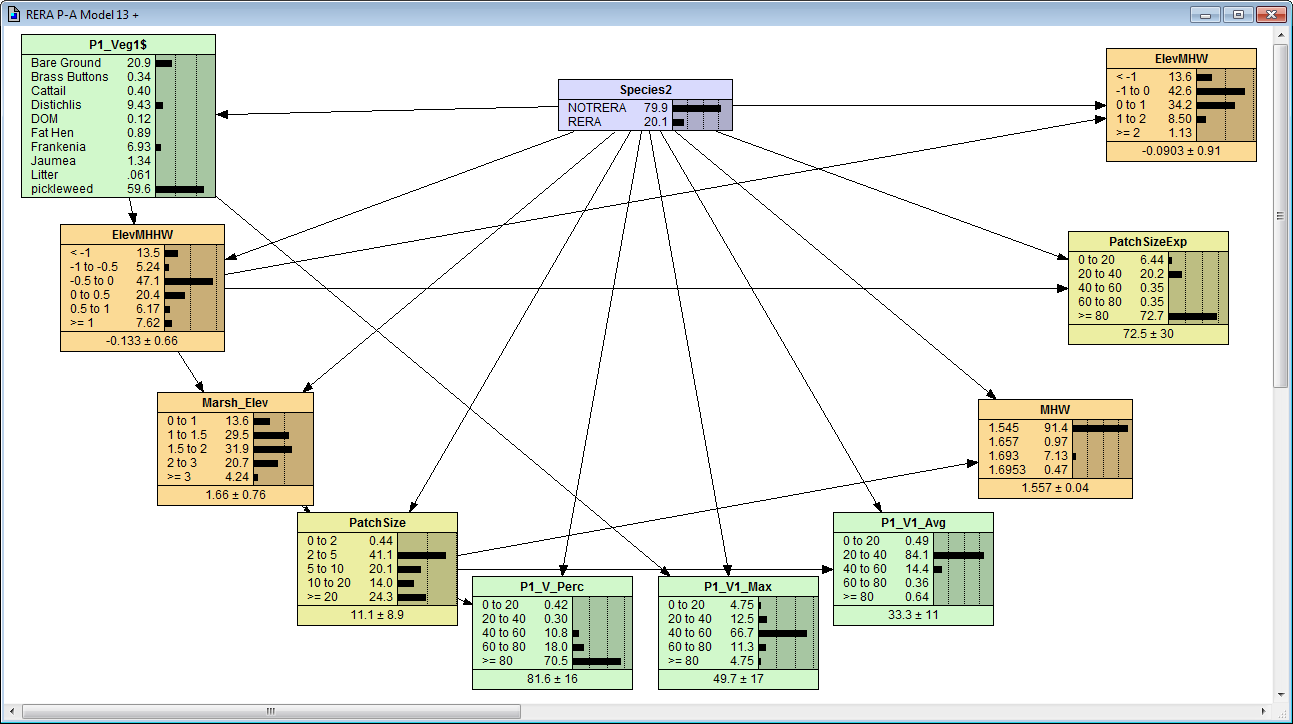


Model no.: 14

Netica model file name: "RERA P-A Model 14.neta"


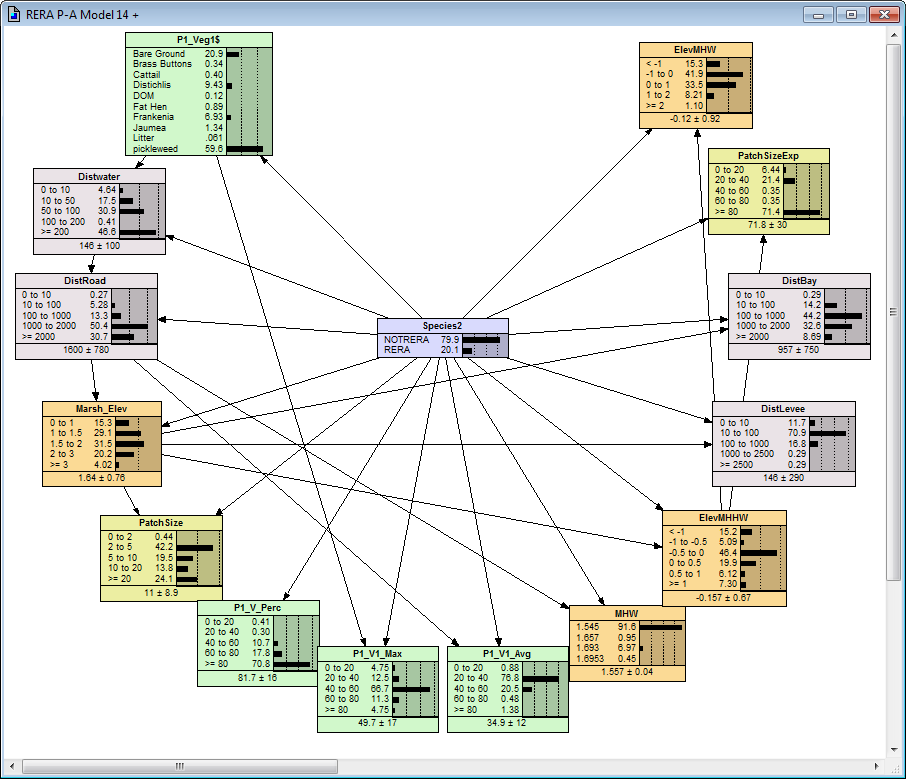


Model no.: 15

Netica model file name: "RERA P-A Model 15.neta"


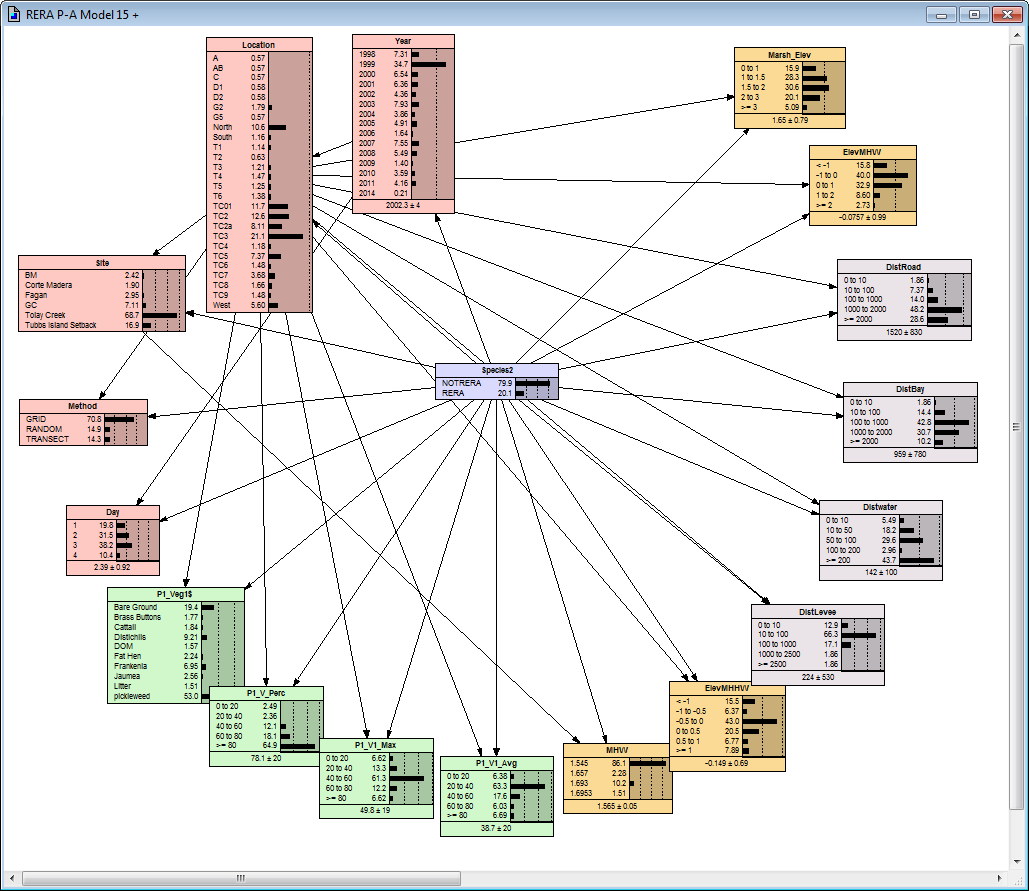


Model no.: 16

Netica model file name: "RERA P-A Model 16.neta"


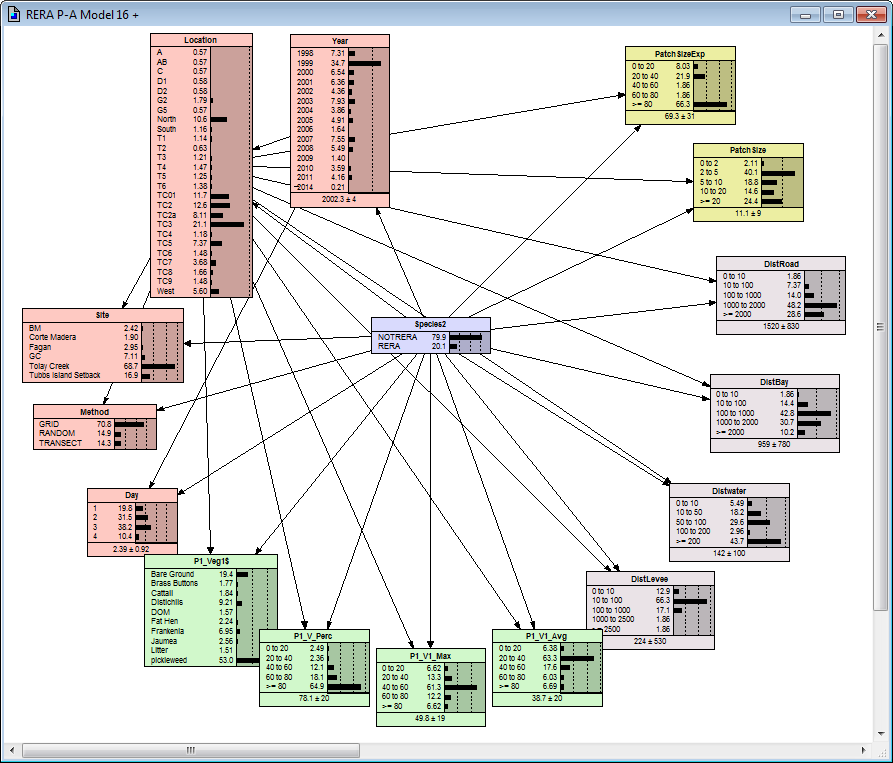


Model no.: 17

Netica model file name: "RERA P-A Model 17.neta"


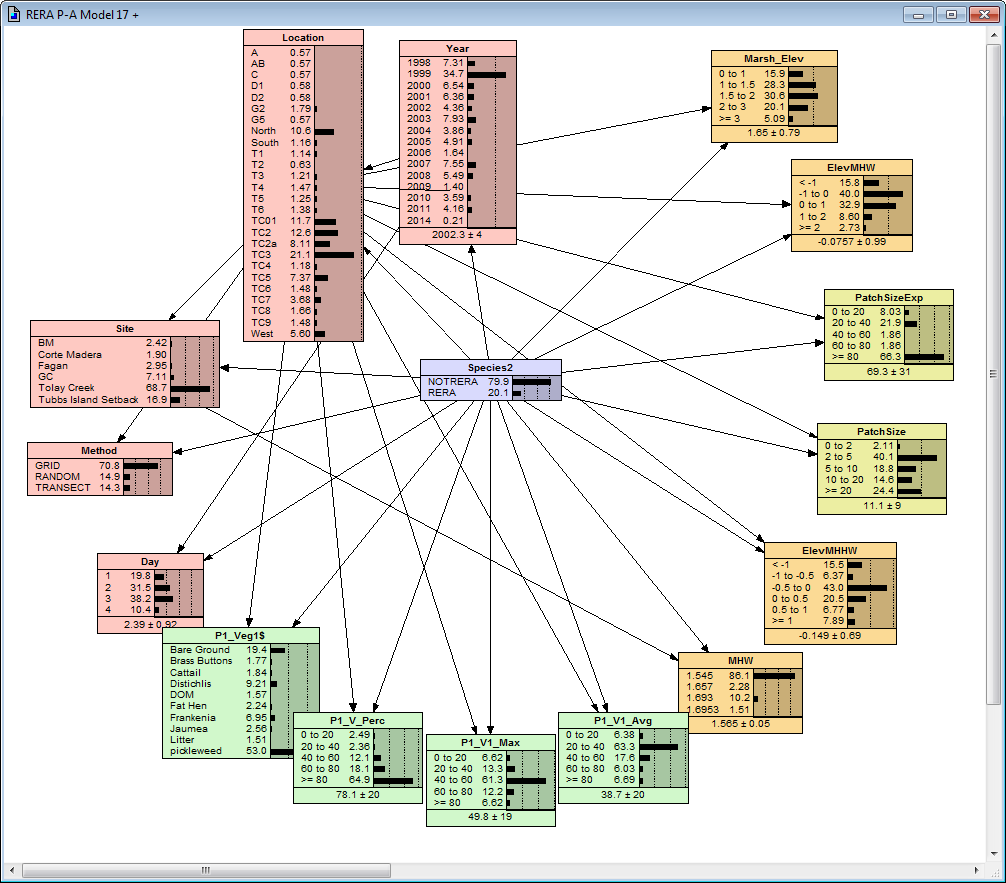


Model no.: 18

Netica model file name: "RERA P-A Model 18.neta"


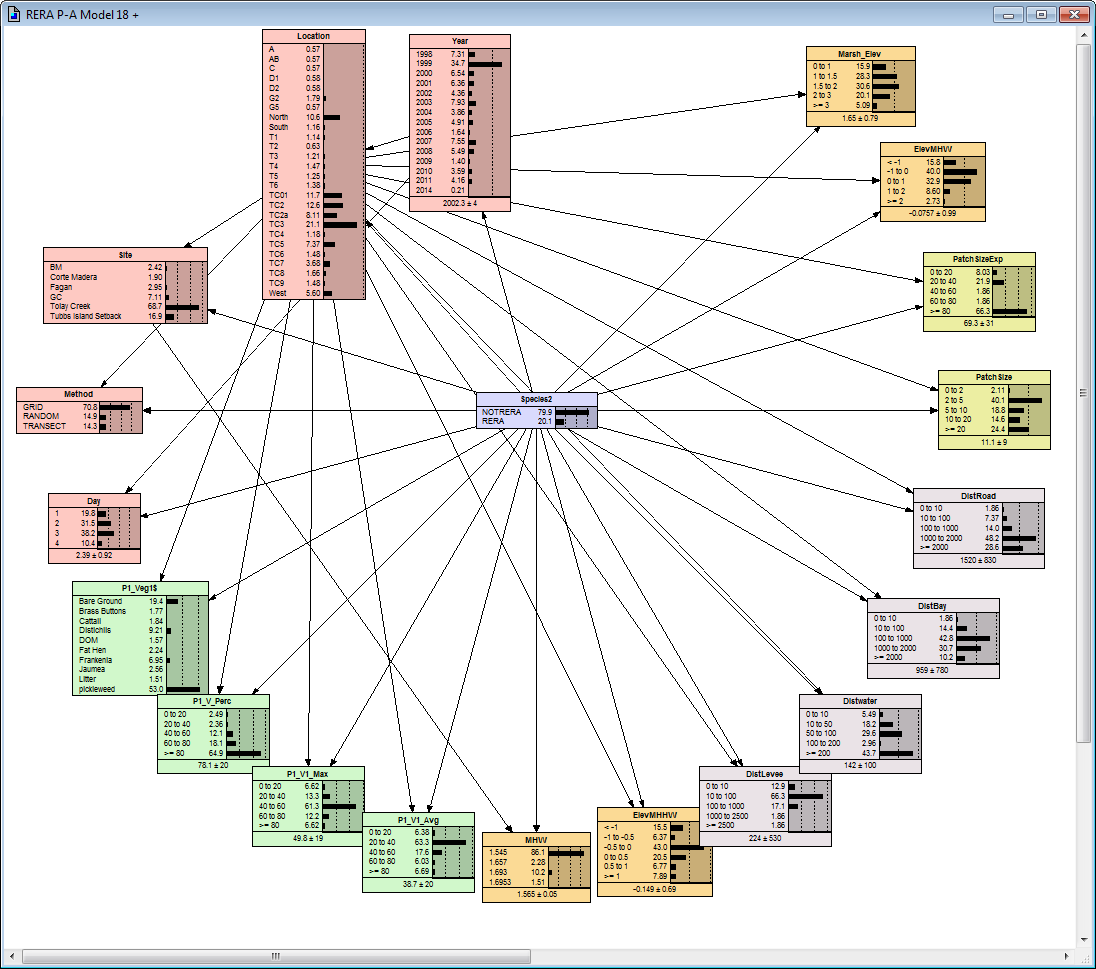


Model no.: 19

Netica model file name: "RERA P-A Model 19.neta"


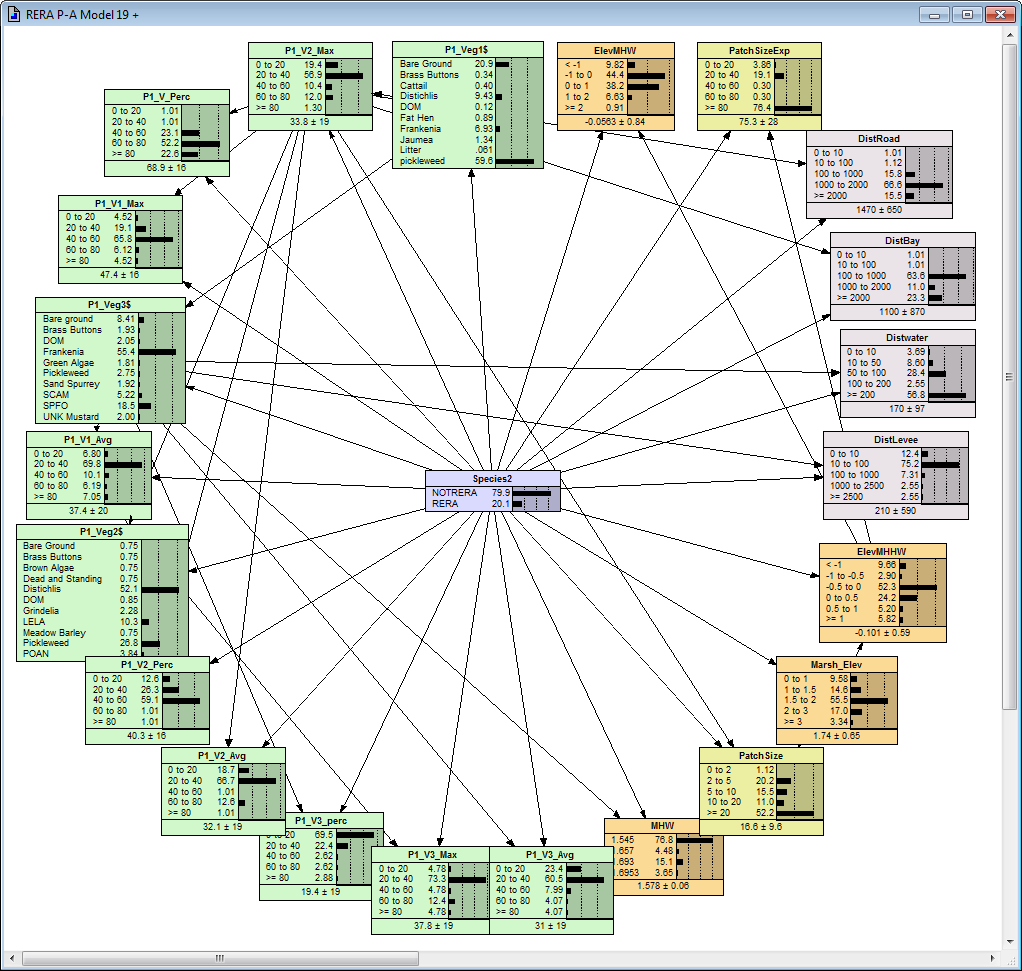


MODEL SET 2 : ALL MAMMAL SPECIES PRESENCE-ABSENCE

Model no.: 20

Netica model file name: "RERA P-A Model 20.neta"


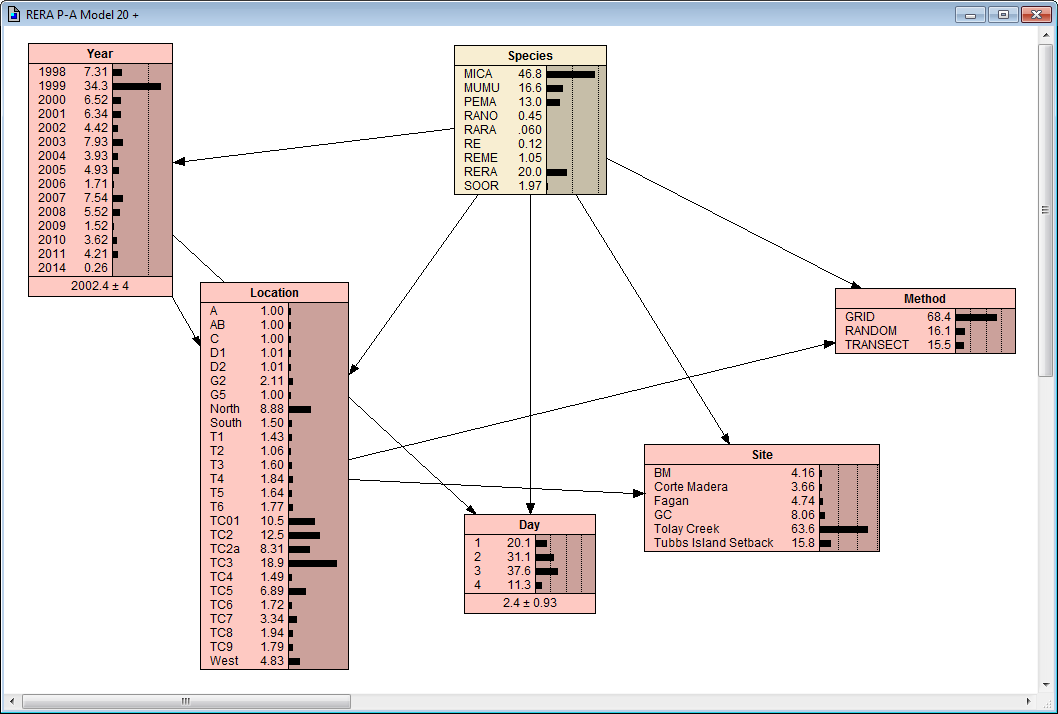


Model no.: 21

Netica model file name: "RERA P-A Model 21.neta"


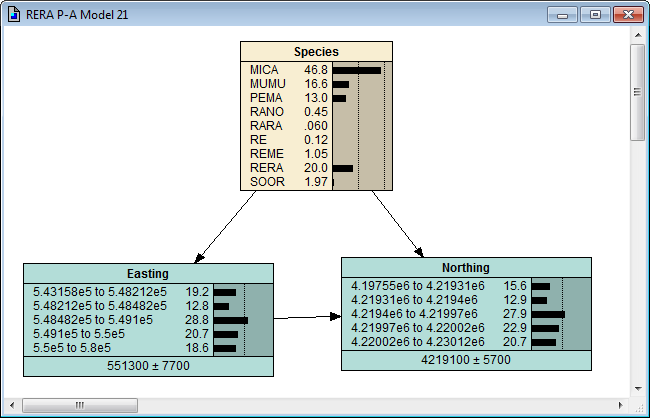


Model no.: 22

Netica model file name: "RERA P-A Model 22.neta"


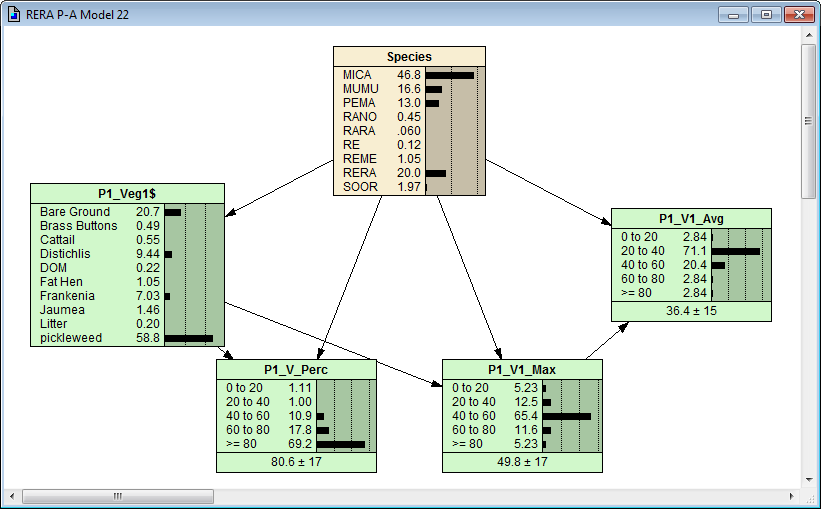


Model no.: 23

Netica model file name: "RERA P-A Model 23.neta"


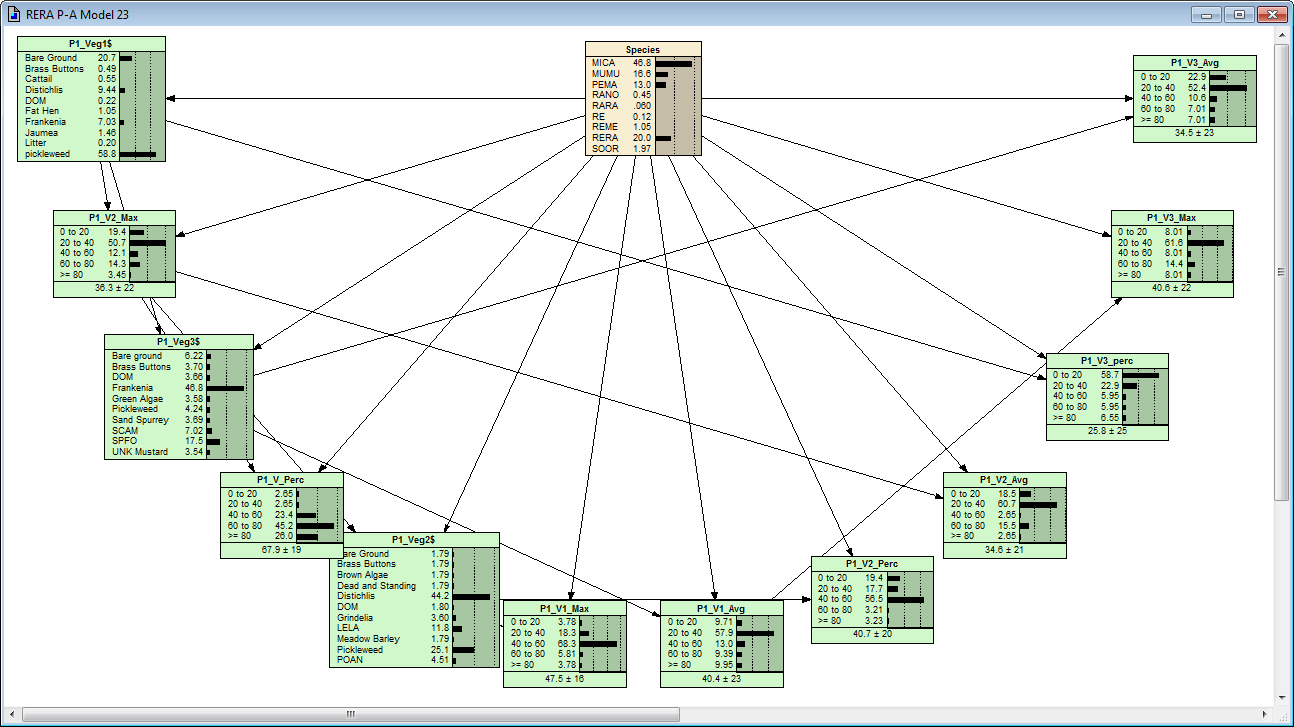


Model no.: 24

Netica model file name: "RERA P-A Model 24.neta"


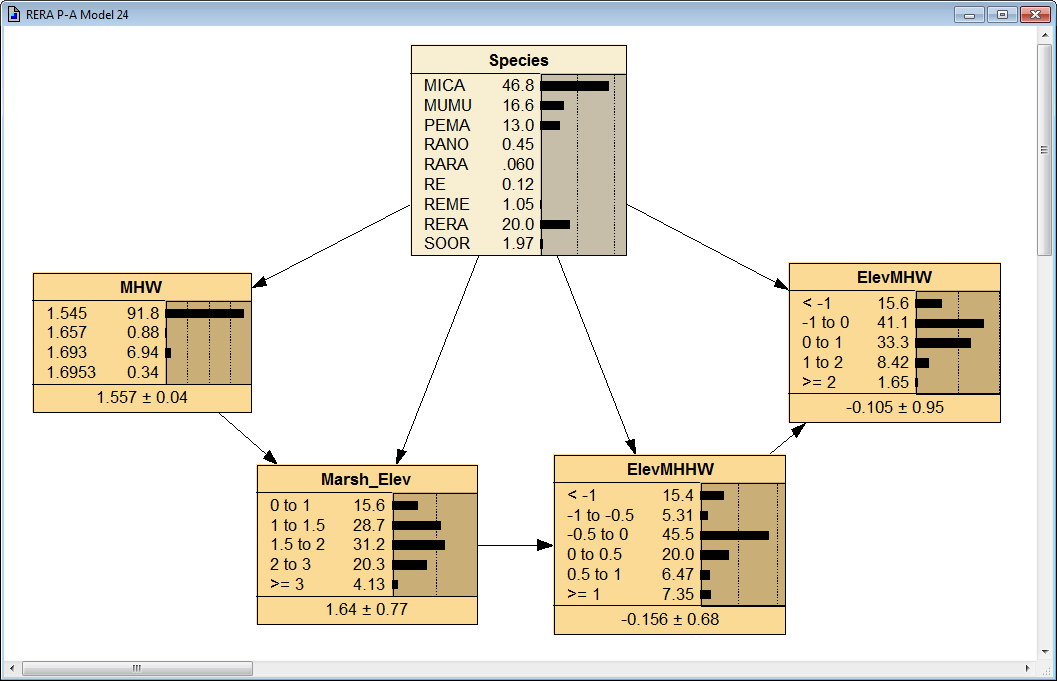


Model no.: 25

Netica model file name: "RERA P-A Model 25.neta"


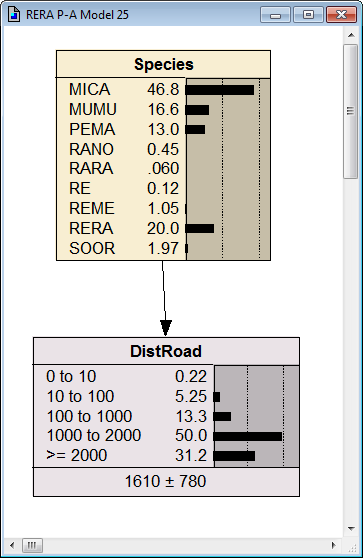


Model no.: 26

Netica model file name: "RERA P-A Model 26.neta"


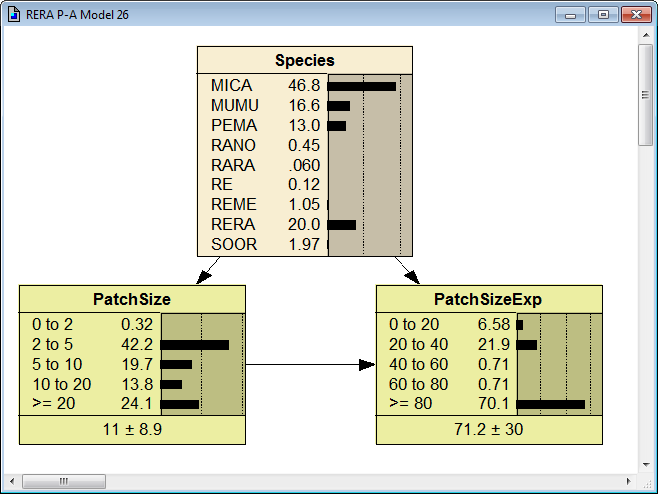


Model no.: 27

Netica model file name: "RERA P-A Model 27.neta"


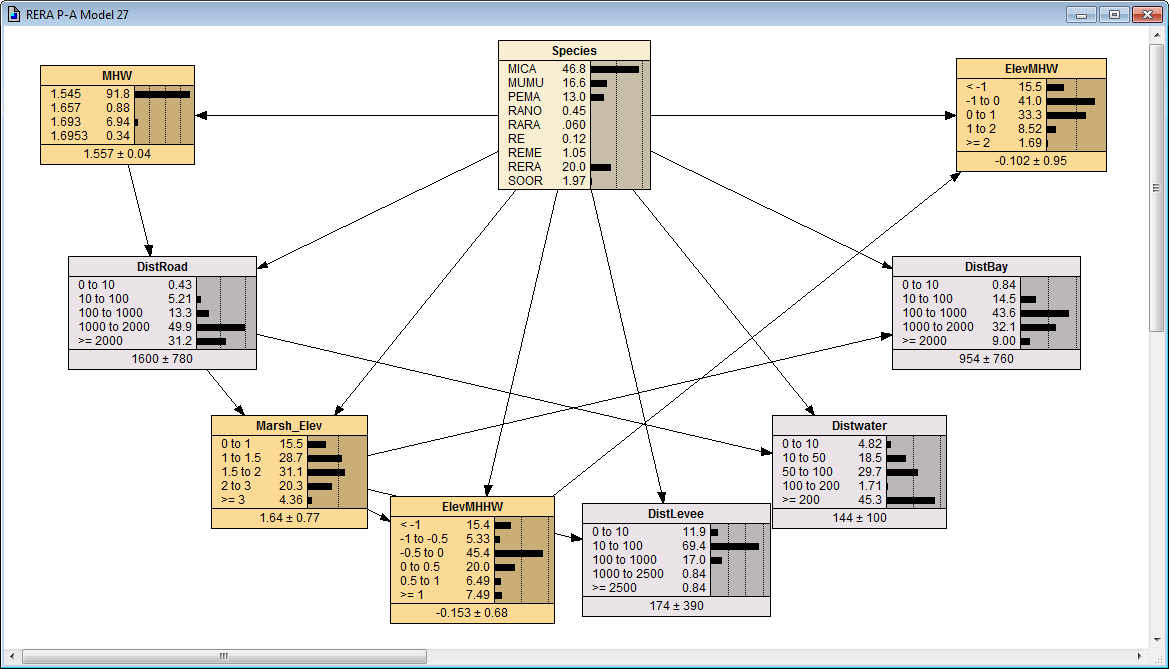


Model no.: 28

Netica model file name: "RERA P-A Model 28.neta"


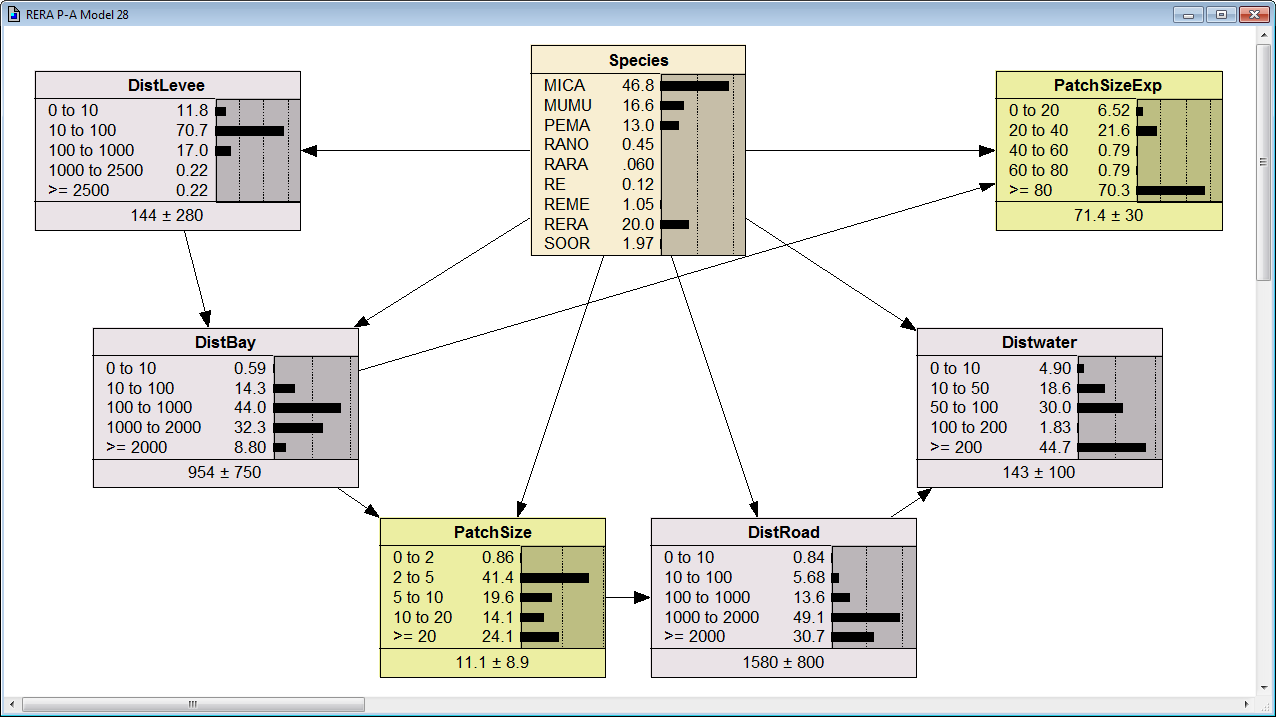


Model no.: 29

Netica model file name: "RERA P-A Model 29.neta"


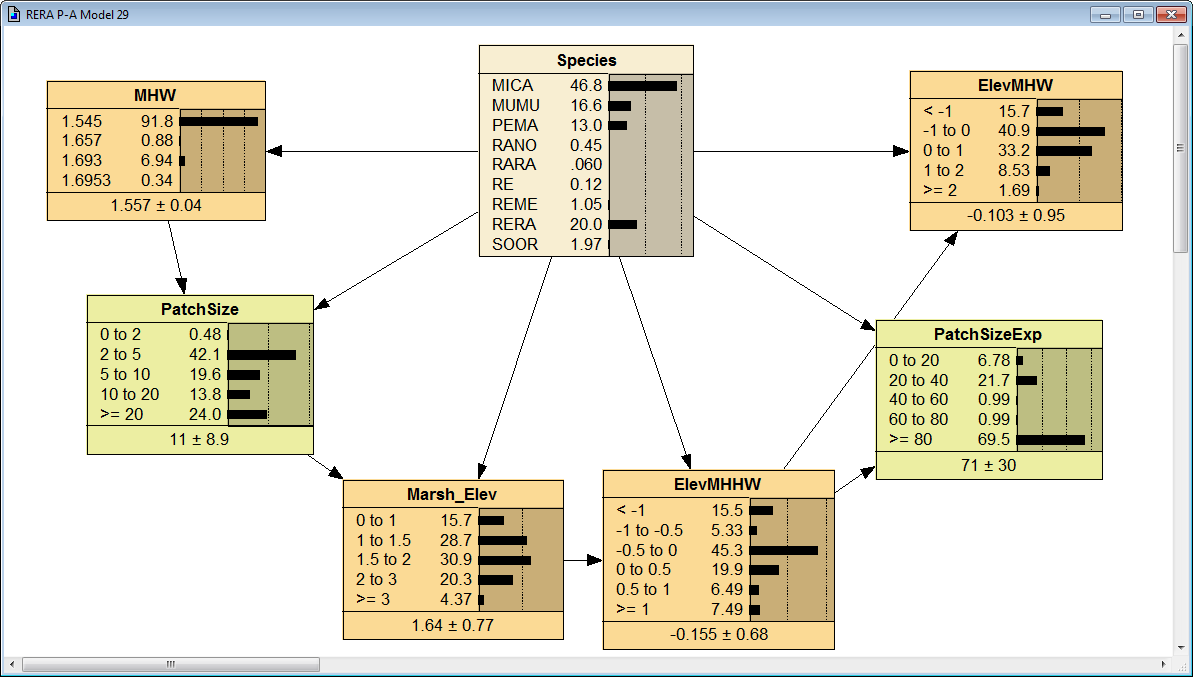


Model no.: 30

Netica model file name: "RERA P-A Model 30.neta"


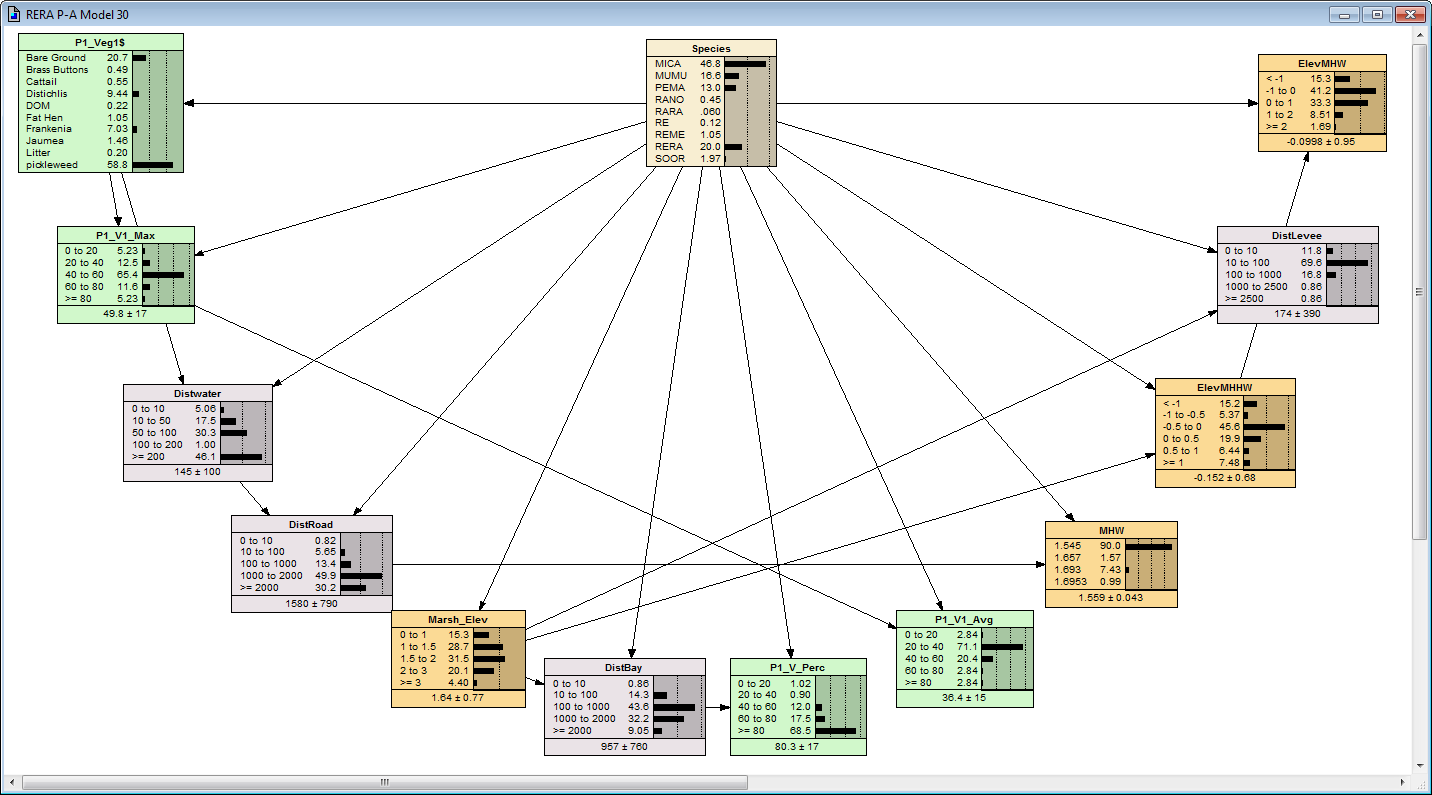


Model no.: 31

Netica model file name: "RERA P-A Model 31.neta"


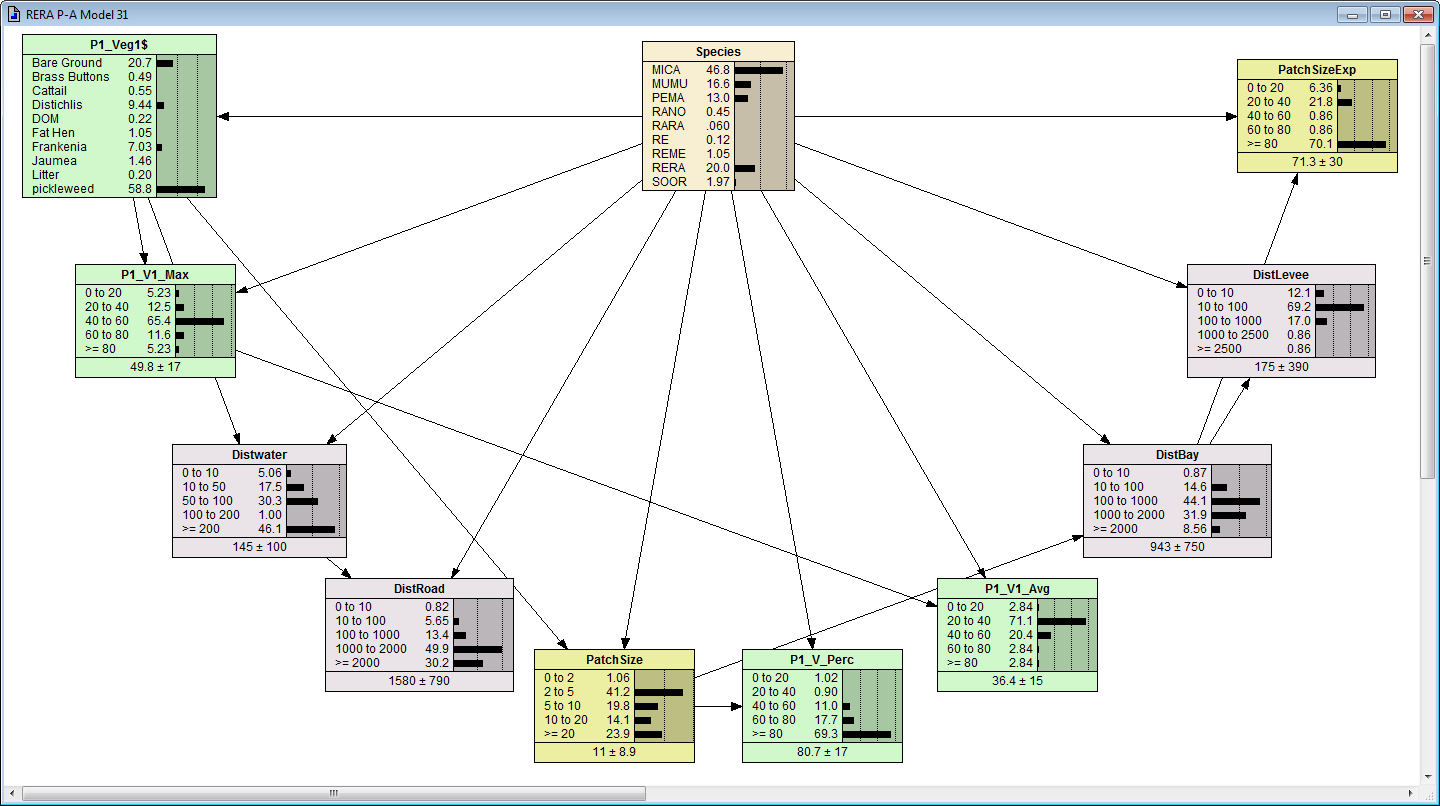


Model no.: 32

Netica model file name: "RERA P-A Model 32.neta"


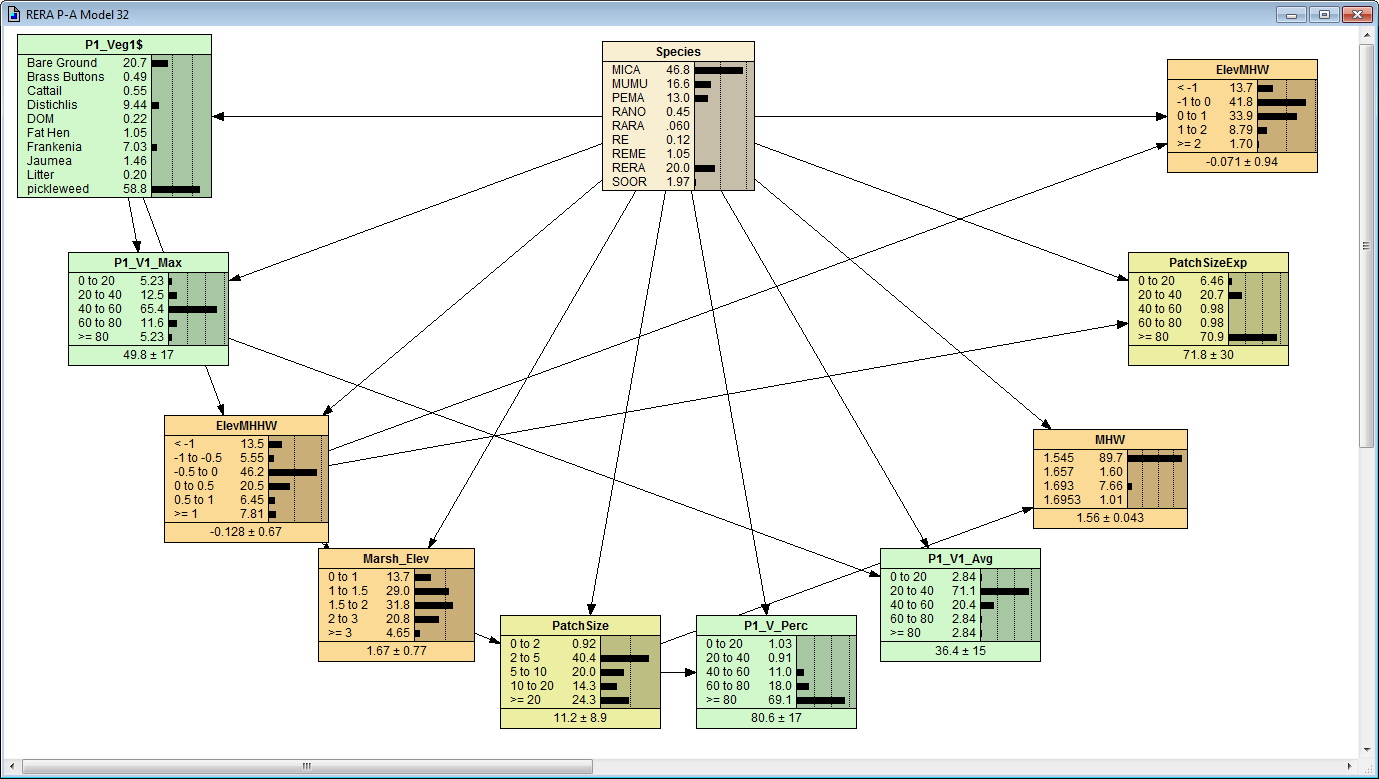


Model no.: 33

Netica model file name: "RERA P-A Model 33.neta"


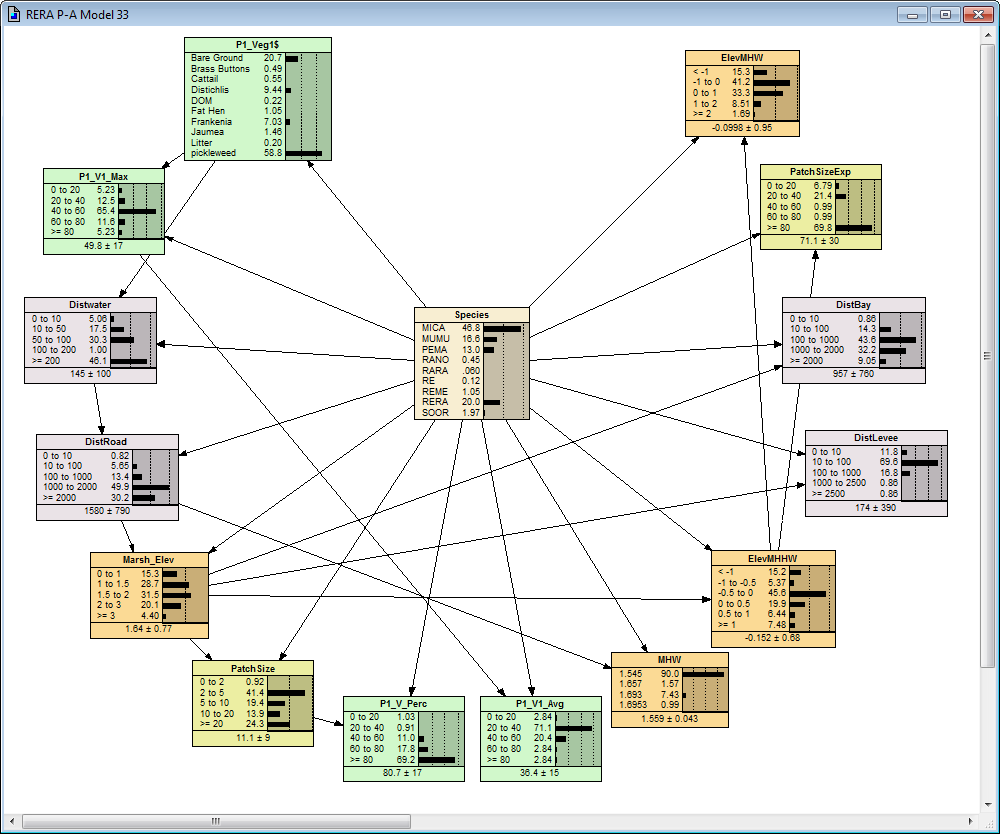


Model no.: 34

Netica model file name: "RERA P-A Model 34.neta"


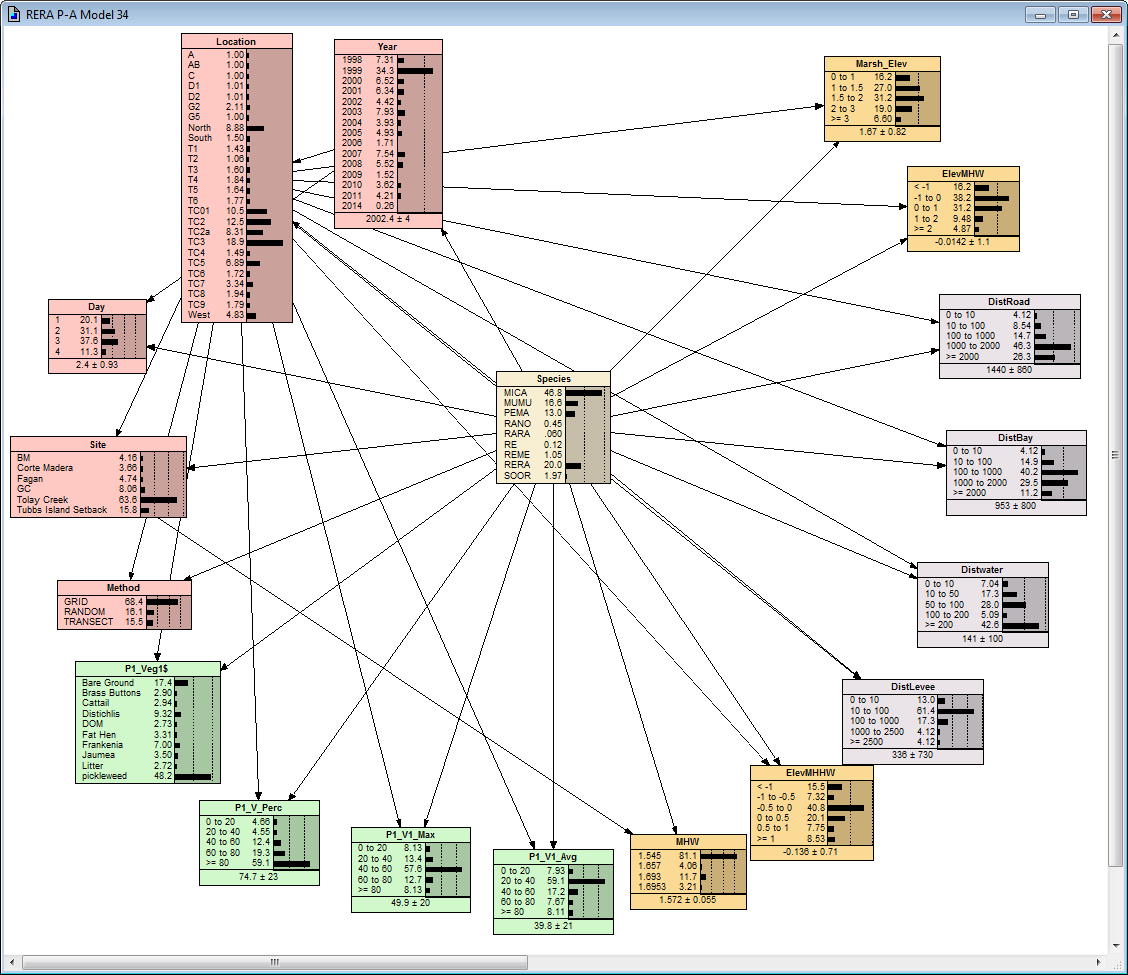


Model no.: 35

Netica model file name: "RERA P-A Model 35.neta"


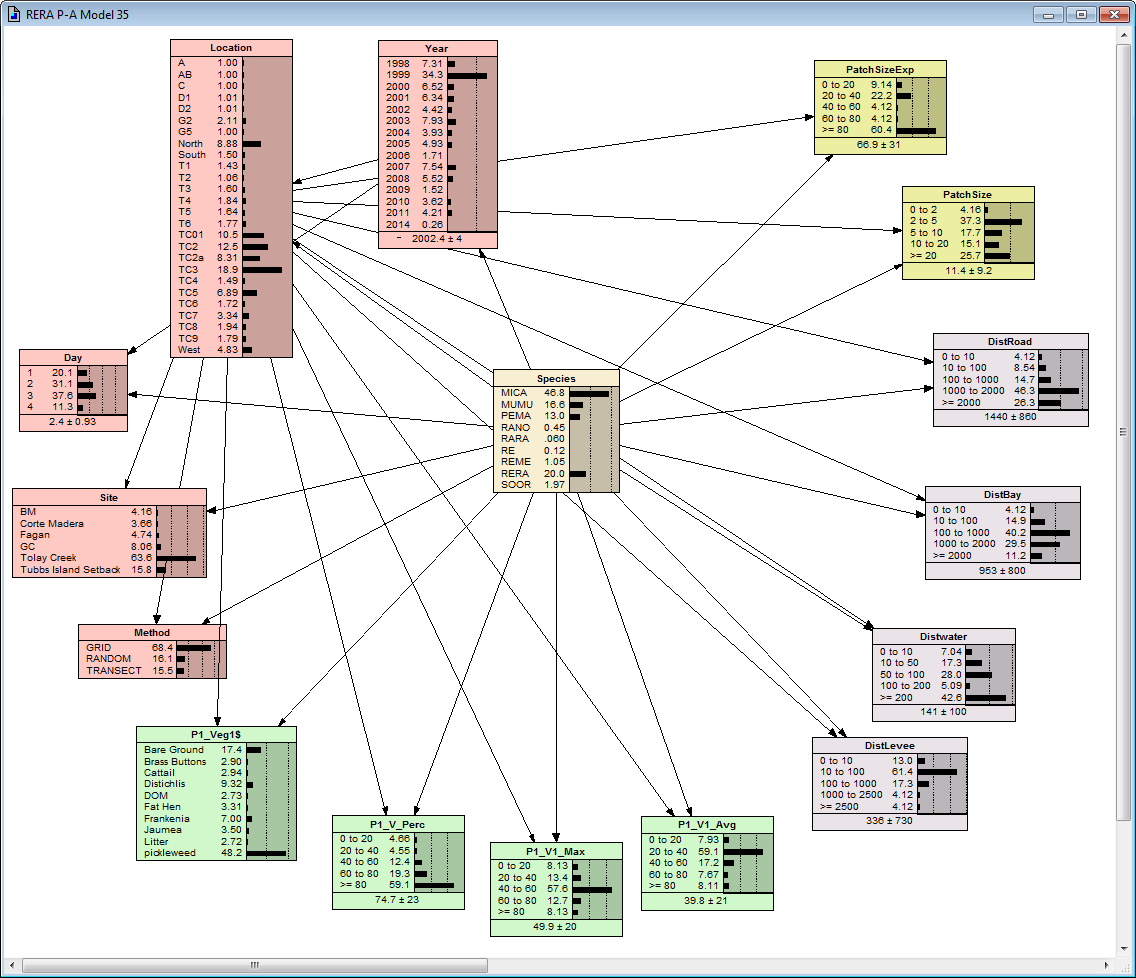


Model no.: 36

Netica model file name: "RERA P-A Model 36.neta"


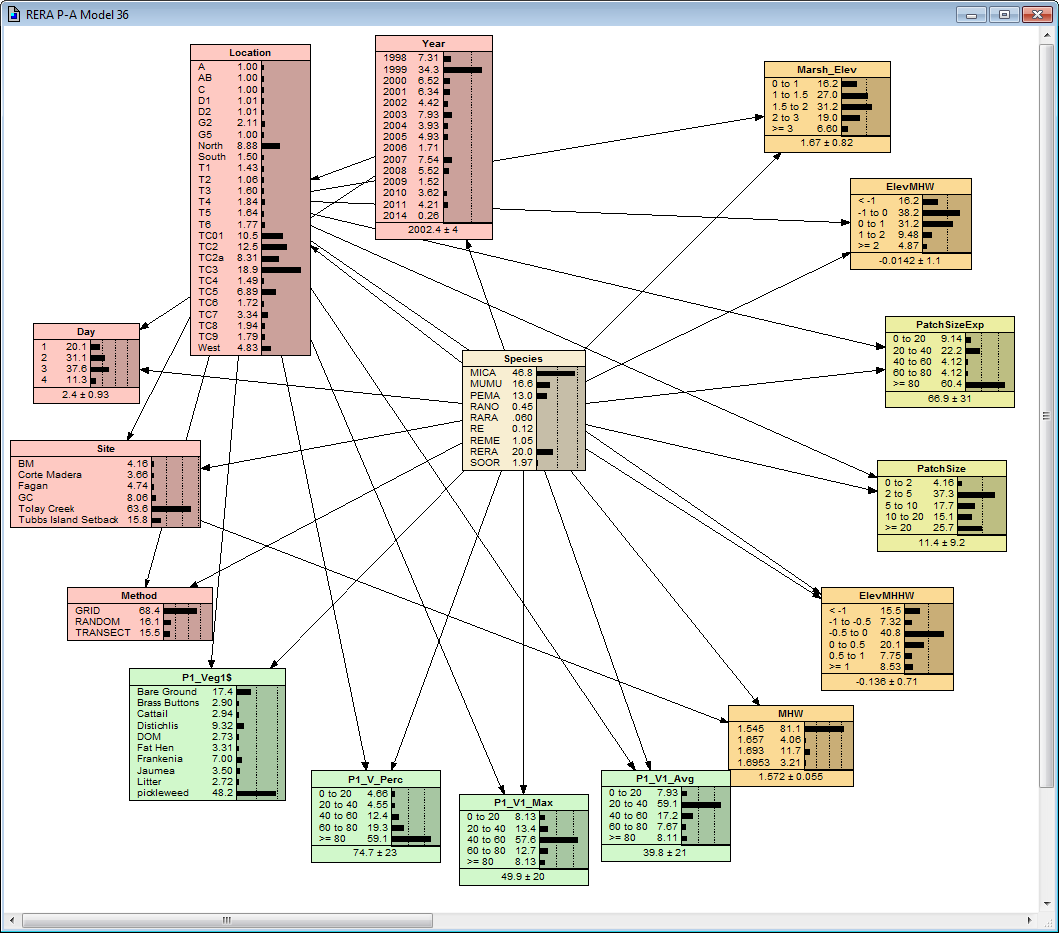


Model no.: 37

Netica model file name: "RERA P-A Model 37.neta"


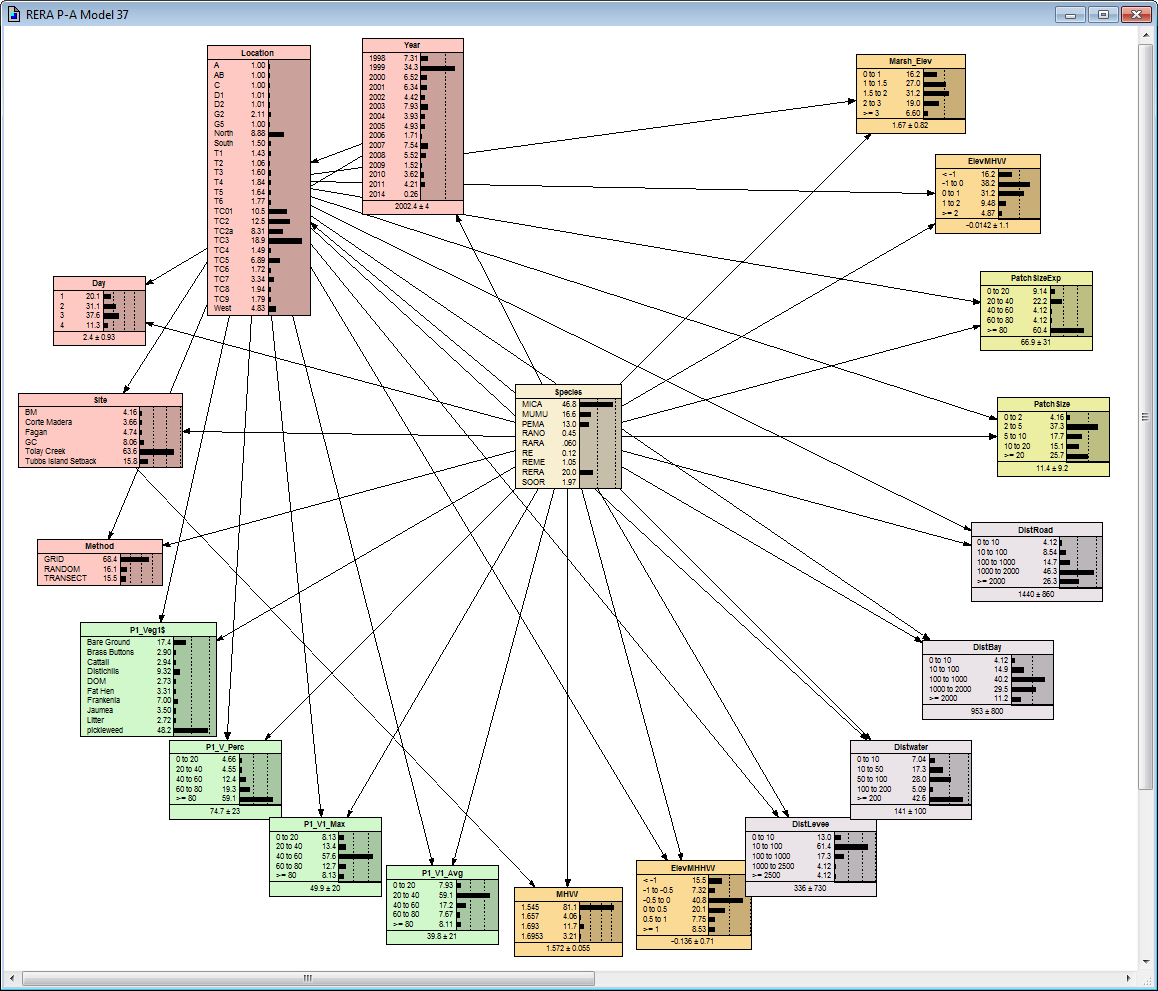


Model no.: 38

Netica model file name: "RERA P-A Model 38.neta"


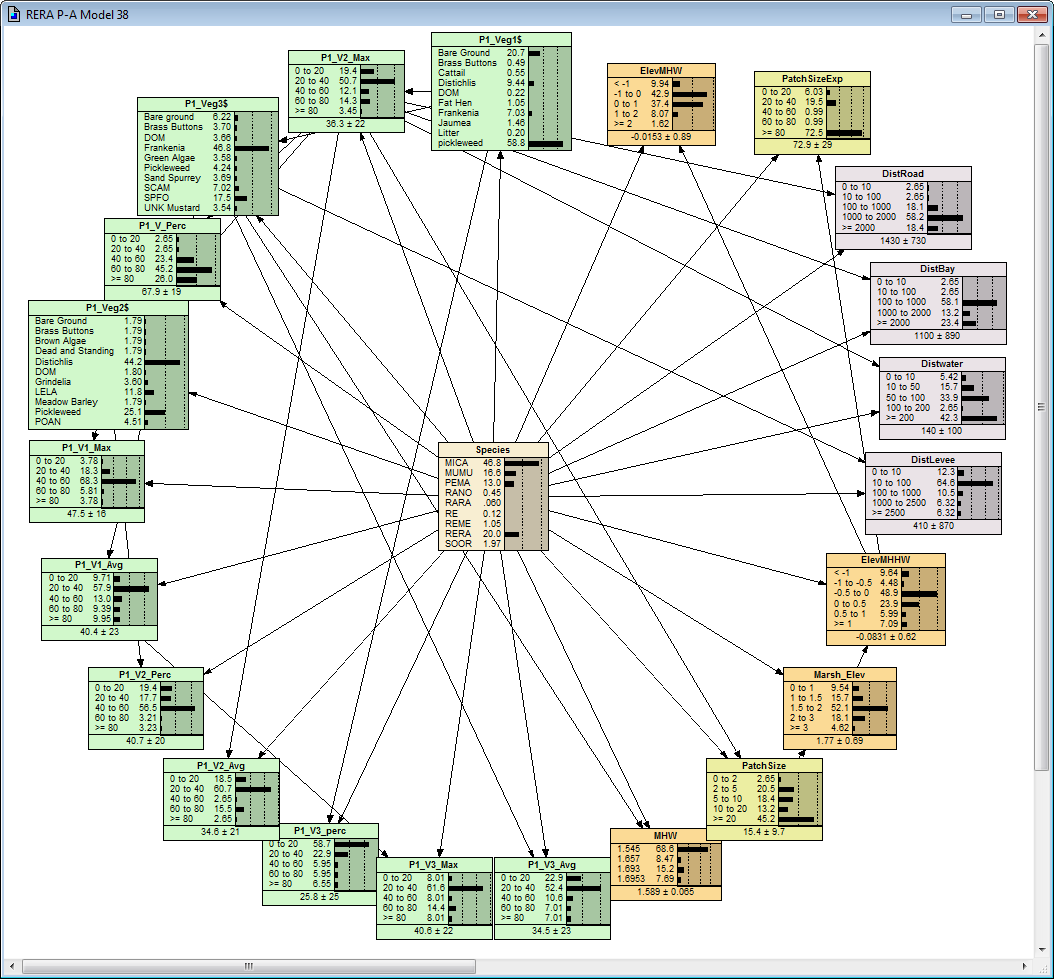

Supplement: Supplementary file 1 [file ECE3-10-662-s001.docx]
